# Blended Psychological Therapy for the Treatment of Psychological Disorders in Adult Patients: Systematic Review and Meta-Analysis

**DOI:** 10.2196/49660

**Published:** 2024-10-29

**Authors:** Kelly Ferrao Nunes-Zlotkowski, Heather L Shepherd, Lisa Beatty, Phyllis Butow, Joanne Margaret Shaw

**Affiliations:** 1 Psycho-Oncology Co-operative Research Group (PoCoG) School of Psychology The University of Sydney Sydney Australia; 2 Centre for Medical Psychology & Evidence-based Decision-making (CeMPED) School of Psychology The University of Sydney Sydney Australia; 3 Susan Wakil School of Nursing and Midwifery Faculty of Medicine and Health The University of Sydney Sydney Australia; 4 Flinders University College of Education, Psychology and Social Work Flinders University Institute of Mental Health and Wellbeing Adelaide Australia

**Keywords:** systematic review, blended psychological therapy, blended care, face-to-face, online, psychological intervention, intervention design, digital care, digital mental health, psychological disorder

## Abstract

**Background:**

Blended therapy (BT) combines digital with face-to-face psychological interventions. BT may improve access to treatment, therapy uptake, and adherence. However, research is scarce on the structure of BT models.

**Objective:**

We synthesized the literature to describe BT models used for the treatment of psychological disorders in adults. We investigated whether BT structure, content, and ratio affected treatment efficacy, uptake, and adherence. We also conducted meta-analyses to examine treatment efficacy in intervention-control dyads and associations between treatment outcomes versus BT model structure.

**Methods:**

PsycINFO, CINAHL, Embase, ProQuest, and MEDLINE databases were searched. Eligibility criteria included articles published in English till March 2023 that described digital and face-to-face elements as part of an intervention plan for treating psychological disorders in adult patients. We developed a coding framework to characterize the BT interventions. A meta-analysis was conducted to calculate effect size (ES; Cohen *d* and 95% CIs) regarding pre- and posttreatment outcomes in depression and anxiety versus BT structure. The review was registered with PROSPERO and followed the PRISMA (Preferred Reporting Items for Systematic Reviews and Meta-Analyses) guidelines.

**Results:**

Searches identified 8436 articles, and data were extracted from 29 studies. BT interventions were analyzed and classified according to mode of interaction between digital and face-to-face components (*integrated* vs *sequential*), role of the components (*core* vs *supplementary*), component delivery (*alternate* vs *case-by-case*), and digital materials assignment mode (*standardized* vs *personalized*). Most BT interventions (n=24) used a cognitive behavioral therapy approach for anxiety or depression treatment. Mean rates of uptake (91%) and adherence (81%) were reported across individual studies. BT interventions were more effective or noninferior to treatment as usual, with large spread in the data and a moderate to large ES in the treatment of depression (n=9; Cohen *d*=–1.1, 95% CI –0.6 to –1.6, *P*<.001, and *z* score=–4.3). A small, nonsignificant ES was found for anxiety outcomes (n=5; Cohen *d*=–0.1, 95% CI –0.3 to 0.05, *P*=.17, and *z* score=–1.4). Higher ESs were found in blended interventions with supplementary design (depression: n=11, Cohen *d*=–0.75, 95% CI –0.56 to –0.95; anxiety: n=8, Cohen *d*=–0.9, 95% CI –0.6 to –1.2); fewer (≤6) face-to-face sessions (depression: n=9, Cohen *d*=–0.7, 95% CI –0.5 to –0.9; anxiety: n=7, Cohen *d*=–0.8, 95% CI –0.3 to –1.3); and a lower ratio (≤50%) of face-to-face versus digital sessions (depression: n=5, Cohen *d*=–0.8, 95% CI –0.6 to –1.1; anxiety: n=4, Cohen *d*=–0.8, 95% CI 0.006 to –1.6).

**Conclusions:**

This study confirmed integrated BT models as feasible to deliver. We found BT to be effective in depression treatment, but anxiety treatment results were nonsignificant. Future studies assessing outcomes across different psychological disorders and therapeutic approaches are required.

**Trial Registration:**

PROSPERO CRD42021258977; https://www.crd.york.ac.uk/prospero/display_record.php?RecordID=258977

## Introduction

### The Impact of Psychological Disorders

Psychological disorders affect approximately 1 billion people globally and were responsible for 10% of the prepandemic global burden of disease in 2019, with estimates of the mental health burden increasing [1]. Depression and anxiety disorders cost US $1 trillion per year globally [2]. Evidence-based, effective mental health care is available, but its provision does not reach everyone who needs it [2,3].

### Digital (or Online) Therapy

Digital psychological therapy provides an opportunity to enhance patient access to psychological treatment [4,5] and is recommended by the World Health Organization as a cornerstone of “comprehensive, integrated and responsive mental health and social care services” [1]. Research regarding digital therapy has largely focused on internet-delivered cognitive behavioral therapy (CBT), demonstrating its efficacy and cost-effectiveness [6,7], particularly in the treatment of depression and anxiety [8-12]. Although uptake and adherence to digital therapy in the research setting have shown improvement [13], engagement is still low, particularly in routine mental health care [14-17]. In addition, research suggests stakeholder preference for face-to-face interventions [18,19]. A systematic review [20] reported on concerns raised by health professionals about the use of digital therapy alone, including perceived nonsuitability for patients due to symptom severity, lack of digital access and literacy, and perception of digital treatment as being less engaging than face-to-face treatments. This review indicated that blended psychological therapy (also called “blended therapy” or “blended care”) was perceived as a midway option between digital and face-to-face therapy.

### Blended Therapy

Blended therapy (BT) is a model of care that combines digital and face-to-face delivery of psychological therapy, integrating benefits from both modalities. Specifically, the face-to-face component is delivered by a mental health professional, such as a psychologist, while the digital component is patient driven [21-23]. Integrating digital therapy with face-to-face interventions in a blended model has the potential to save professionals’ and patients’ time (eg, transport to and from the clinic); increase the frequency of sessions; improve treatment uptake, adherence, and maintenance; and boost therapy effects [24-26].

A systematic review by Erbe et al [24] (N=44) found that BT may improve dropout rates and save health professionals’ time compared with exclusively face-to-face interventions. Despite increasing evidence of the benefits of blended psychological therapy for patients [22,24], there is a lack of research specifically focused on “what, how, where, and when” BT is effective to inform future BT interventions [21,22,27]. The rationale for our systematic review emerges from the scarcity of data specifically focusing on BT processes including BT content and structure, which hinders scientific reproducibility of BT and impacts its implementation success.

### Objectives of This Review

Seeking to address these gaps in BT literature, our systematic review and meta-analysis expands on the work of Erbe et al [24] and aims to (1) identify and describe the structure, content, and ratio of the face-to-face and digital components in BT interventions applied for the treatment of psychological disorders and (2) investigate whether there is an association among the structure, content, and ratio of blended components and the treatment efficacy of, uptake of, and adherence to BT.

## Methods

### Design

This review followed the PRISMA (Preferred Reporting Items for Systematic Reviews and Meta-Analyses) [28] guidelines and was registered with PROSPERO (CRD42021258977). The PRISMA checklist is provided in Multimedia Appendix 1.

### Search Strategy

The PsycINFO, CINAHL, Embase, ProQuest, and MEDLINE databases were searched using keywords and Medical Subject Headings terms—“blended”; “online”; “face-to-face”; “treatment”; “therapy”; “care”; “mental disorders”; “psychological distress”; and “psychological disease”—for articles published in English (Multimedia Appendix 2). The search included articles published till May 2022, and an updated search was conducted in March 2023. Reference lists of the included studies were also manually searched.

### Study Selection

#### Inclusion and Exclusion Criteria

Studies that described or applied an intervention where both digital and face-to-face elements were integrated or delivered sequentially were included. We included studies in which the participants were aged ≥18 years and diagnosed with a psychological disorder. Studies solely investigating populations other than this target group (health care professionals, student cohorts, employees, etc) were excluded.

#### Comparators

The comparator or control groups included treatment as usual (pharmacological or psychological intervention and standard medical care), waitlist, or other interventions.

#### Data Abstracted

The primary focus was the intervention design, including descriptions of the structure, content, and ratio of the sessions used in each model. Secondary outcomes were (1) a psychological therapy approach used in the BT models, (2) patient groups for which BT was applied, (3) treatment efficacy, (4) uptake and adherence, (5) health service outcomes (eg, cost-effectiveness), (6) patients’ acceptability of BT, (7) therapeutic alliance rates, and (8) barriers and facilitators reported.

#### Article Screening and Selection

All search results were uploaded into Covidence software [29]. Two reviewers (KFN-Z and JMS) screened the titles and abstracts independently. Full-text reviews based on the eligibility criteria were conducted by KFN-Z and PB or JMS.

### Data Extraction

Data extraction was conducted by KFN-Z using a purpose-designed data extraction template. Extraction results were partially reviewed (6/29, 20%) by a second coder (JMS) to assess accuracy. To capture any missing data, the corresponding authors were e-mailed twice. Data extracted included the following: (1) study characteristics—authors, year of publication, country, study setting, study aims, study type, sample size, control group (where applicable), therapy approach applied, primary or secondary psychological outcomes, and symptom assessment measures and participant characteristics such as age, sex, diagnosed psychological disorders, severity of symptoms, and individual study outcomes; (2) intervention characteristics—BT intervention design based on the structure, content, and ratio of BT sessions; number, periodicity, and duration (in minutes) of face-to-face and digital sessions; treatment length (in weeks); and (3) BT intervention outcomes—treatment efficacy, uptake, adherence, cost-effectiveness, acceptability, therapeutic alliance, and barriers and facilitators to BT reported.

### Data Analysis

To address the objectives of this review, quantitative variables regarding the BT intervention structure were summarized and described. We used descriptive statistics (mean, percentages, and range) to describe quantitative data regarding study and participant characteristics; BT intervention uptake, adherence, and completion rates; treatment length (in weeks); number, time (in minutes), ratio, and periodicity of face-to-face and digital sessions; treatment acceptability; efficacy; and therapeutic alliance. Barriers to and facilitators of BT were qualitatively analyzed using a thematic analysis [30] approach. Qualitative data on BT structure and content were analyzed using a content analysis approach [31]. Categories and subcategories were summarized in a framework that builds on the concepts described by Erbe et al [24] (Textbox 1).

**Classification—blended model designs.**

**Interaction between face-to-face and digital components: *integrated* vs *sequential***

- *Integrated* models present both the digital and face-to-face components as collaborating parts within a therapy regimen, with both components delivered within the course of the intervention [24].
- *Sequential* models present the digital component delivered in entirety before or after face-to-face component delivery [24]. Sequential interventions start by delivering a “batch” of either face-to-face or digital sessions. Once the first “batch” is finished, the other component gets delivered.
  - *Stepped care* is considered a special type of *sequential* design in which the digital component is a step in the intervention sequence [24]. *Stepped care* interventions deliver the least intensive or costly treatment first and then progress to more intensive or aftercare treatment, if required. Hence, the “blend” in stepped care only effectuates after the first stage (digital) of treatment is complete and if patients require additional (face-to-face) care.

**Role of the components in the intervention: *core* vs *supplementary***

- *Core* components are an indispensable part of the blended intervention, as they deliver new therapeutic elements (ie, modules complement each other).
- *Supplementary* components present content that has already been discussed during the intervention, that is, content of one component is supplementary to the content delivered in the main component. For example, face-to-face content may be supplemented by reinforcing exercises and homework on the web.

**Delivery pattern of face-to-face or digital components: *alternate* vs *case by case***

- *Alternate* delivery is a configuration in which each session is delivered by alternating face-to-face or digital components in a fixed ratio. The distribution of components is preset for the entire intervention; this may feature as a 1:1 ratio, but other ratios (eg, 2 digital to1 face-to-face) of distribution are possible.
  - *Linear* delivery is specific to *sequential* designs in which all digital sessions are delivered in a row followed by all face-to-face sessions in a row, or vice versa.
- *Case-by-case* delivery is a configuration in which therapists assess and formulate a strategy for distributing the face-to-face or digital sessions adapted to the clients’ or patients’ needs on a case-by-case basis.

**Digital content assignment: *personalized* vs *standardized***

- *Personalized* content assignment is not preset; therapists and patients decide which materials to complete, tailoring them to patients’ needs.
- *Standardized* content assignment is largely preset; materials are delivered to all patients undergoing treatment with little or no changes to content.

#### Meta-Analyses

Although a meta-analysis was not originally included in the registered protocol, data collection and analysis processes indicated the relevance of meta-analyzing treatment outcomes for enhancing systematic review results. The meta-analysis was conducted using the Comprehensive Meta-Analysis program [32] to investigate treatment efficacy between the treatment and control dyads. A meta-analysis of *BT interventions only* was also conducted to investigate associations between BT structure and content and treatment outcomes. Pre-post outcome means and SD, alongside data on sample size at post–time points were included in the analysis. Standardized difference in means (Cohen *d*) and 95% CIs were used as effect size (ES) measures, and *z* values were used to test the null hypothesis (ES=0). We used a random effects meta-analysis due to expected heterogeneity. ES was set to negative numbers to show the change in symptoms (the lower the number, the higher the reduction in symptoms). Heterogeneity was assessed using *Q* test (*Q*), I-squared (*I^2^*), Tau-squared (T^2^), and Tau (T) scores. Publication bias was assessed using funnel plots and the trim and fill method by Duval and Tweedie [33].

#### Risk of Bias and Quality Assessment

Two independent reviewers (KFN-Z and JMS) assessed the risk of bias using the National Institutes of Health Study Quality Assessment Tool (quantitative) [34], the Critical Appraisal Skills Programme qualitative checklist [35], or the Mixed Methods Appraisal Tool [36].

## Results

### Database Search Results

**Figure 1:**
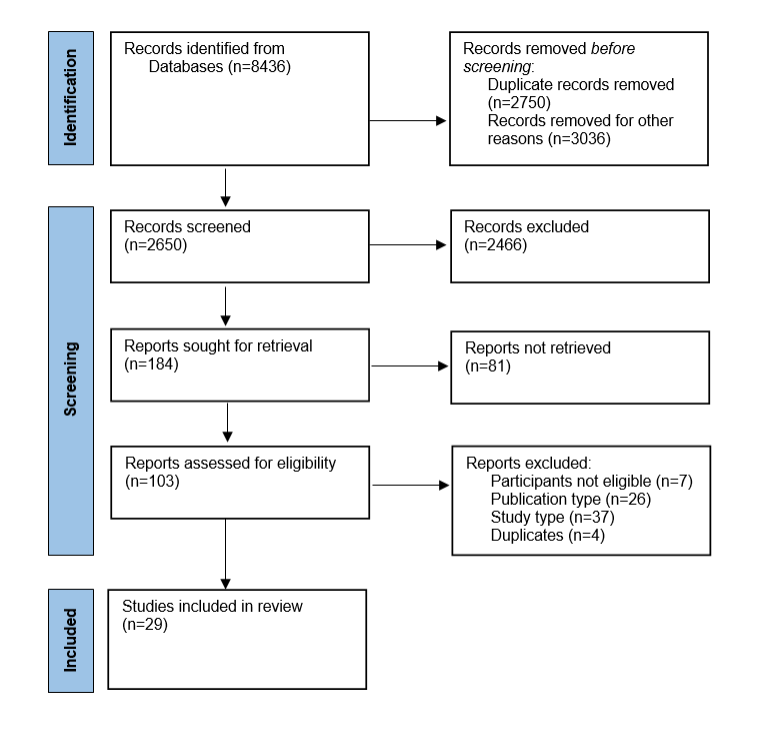
PRISMA (Preferred Reporting Items for Systematic Reviews and Meta-Analyses) 2020 flow diagram.

Database searches identified 2650 papers after removal of duplicates. Title and abstract screening resulted in 103 articles for full-text review. A total of 30 eligible articles were identified but only 29 were included in the review—one eligible paper was excluded as it reported the same data. The PRISMA diagram in Figure 1 [28] provides the details of the process.

### Quality Assessment

Quality assessment deemed most studies (n=22) to be of “good quality” as they addressed most of the quality assessment criteria applied. Seven articles [23,37-42] were classified to be of “fair” quality (Multimedia Appendix 3).

### Study Characteristics

Of the 29 articles, 25 (86%) were prospective studies (randomized controlled trials [RCTs], n=14; feasibility or pilot, n=4; cohort or single arm, n=7); 3 (10%) were retrospective analyses of cohort studies; and 1 (3%) study used qualitative methods only. Most BT interventions (22/29, 76%) primarily treated depression, either exclusively (13/29, 45%) or in combination with anxiety treatment (9/29, 31%). Most studies (28/29, 96%) used a CBT approach, with 3 (10%) combining CBT and other approaches such as dialectical behavioral therapy and acceptance and commitment therapy (n=2) and motivational interviewing (n=1). Outcomes assessed were primarily symptom reduction (25/29, 86%), although 4 (14%) studies reported on the intervention process or working alliance outcomes as the primary focus (Table 1).

Collectively, the studies included a total of 12,322 (range 3-4448) patient participants, with 57.71% (n=7111) receiving BT interventions. Of the studies reporting on symptom reduction (28/29, 96%), all prescreened for clinical levels of psychological morbidity (Table 2).

**Table 1:** Characteristics of reviewed studies.

Study; country	Study setting	Study aims	Study design	Participants, N; BT^a^ group, n; comparator, n	Therapy type; clinical outcomes
Askjer and Mathiasen [43], 2021 and Mathiasen et al [44],^b^ 2022; Denmark	Specialized mental health care	Explore if WA^c^ predicted treatment outcomes	Exploratory; secondary analysis from an RCT^d^	76; 38; 38; comparator: F2F^e^ CBT^f^	CBT; WA, depressive symptoms
Berger et al [45], 2018; Germany	Routine outpatient psychotherapy practices	Investigate web-based treatment as adjunctive to depression treatment vs regular psychotherapy	Two-armed, pragmatic RCT	98; 51; 47; comparator: TAU^g^: psychotherapy	CBT; depressive symptoms
Bisson et al [46], 2022; United Kingdom	Primary and secondary mental health settings	Determine if CBT-TF^h^ was noninferior to F2F CBT-TF for PTSD^i^	Pragmatic, multicenter, noninferiority RCT	196; 97; 99; comparator: F2F CBT-TF	CBT; severity of symptoms of PTSD
Cloitre et al [47], 2022; United States	Routine care in mental health outpatient clinics	Assess 2 ratios of F2F sessions to self-guided work on trauma-exposed veterans	Quasi-experiment with a noninferiority design	202; 202; NC^j^	Transdiagnostic, trauma-informed CBT; PTSD and depression
Duffy et al [48], 2020; England	Specialized mental health care service	Investigate iCBT^k^ as a prequel for high-intensity depression and anxiety treatment	Uncontrolled feasibility design (open study)	123; 123; NC	CBT; anxiety and depression and work and social functioning
Etzelmueller et al [49], 2018; Germany	Routine care practice (clinics)	Evaluate patient experience of a blended iCBT service	Qualitative, semistructured interviews	15; 15; NC	CBT; ND^l^ depression
Høifødt et al [37], 2013; Norway	University outpatient clinic	Evaluate effectiveness and acceptability of a guided web-based program for depression	RCT	106; 52; 54; comparator: delayed treatment, waitlist, or TAU	CBT; depression symptoms
Jacmon et al [42], 2009; Australia	Psychology private practice	Assess cost-effectiveness and convenience of partially digital depression treatment	Single-arm, pretest-posttest study	9; 9; NC	CBT; depression levels
Kemmeren et al [50], 2019; France, Germany, Poland, and Netherlands	Multisetting (specialized mental health and routine primary care)	Examine use of and engagement to blended CBT for depression	Exploratory, secondary study from RCT	231; 231; NC	CBT; ND depression
Kenter et al [51], 2013; Netherlands	Mental health care center; routine care	Report on the uptake of digital treatment, on the profile of patients who prefer digital therapy, and on symptom reduction vs waitlist	Observational study (electronic patient database)	104; 55; 49; comparator: waitlist	PST^m^; ND depression, anxiety, and burnout
Kenter et al [52], 2015; Netherlands	Mental health service	Compare the effects and costs between blended and F2F treatments	Naturalistic study: examined records of patients	4448; 168; 4280; comparator: TAU: F2F	CBT; ND depression and anxiety
Kok et al [38], 2014; Netherlands	Outpatient clinics	Assess clinical effectiveness of internet-based guided self-help vs waitlist	RCT	212; 105; 107; comparator: waitlist	Psychotherapy; phobia and avoidance behavior
Kooistra et al [23], 2016; Netherlands	Outpatient specialized mental health care center	Develop and evaluate a structured, blended CBT protocol for patients with depression	Focus-groups and single-arm, pre-post	30; 9; NC (12 patients)	CBT; ND depression
Kooistra et al [26], 2019; Netherlands	Specialized mental health care (outpatient services)	Compare costs and effectiveness of blended vs standard CBT for depression	Pilot RCT with 2 parallel groups	102; 53; 49; comparator: CBT-F2F	CBT; self-reported depression severity
Kooistra et al [53], 2020; Netherlands	Outpatient specialized mental health clinics	Investigate WA in bCBT^n^ for depression	Exploratory, secondary study from pilot RCT	92; 47; 45; comparator: regular CBT	CBT; ND depression levels
Lungu et al [54], 2020; United States	Employer program	Evaluate the effectiveness of a video-based CBT and internet intervention	Retrospective cohort study	385; 385; NC	CBT+UTP^o^, ACT^p^, and DBT^q^; ND depression and anxiety
Ly et al [55], 2015; Sweden	Clinical setting	Evaluate a blended treatment for depression	Noninferiority RCT	93; 46; 47; comparator: F2F BA^r^	BA; depression
Månsson et al [39], 2013; Sweden	Clinical setting	Explore clinical outcomes and user experiences of internet-delivered therapy. To develop or test a bCBT model	Mixed methods, case series, pilot study (pre-post re BT testing)	23; 15; NC (patients, n=15; therapists, n=8)	CBT; ND anxiety and depression
Månsson et al [40], 2017; Sweden	Outpatient psychiatric clinic	Evaluate an internet-based support as adjunct to F2F CBT	Feasibility study	54; 45; NC (patients, n=45; therapists, n=9)	CBT; ND anxiety and depression symptoms
Mol et al [56], 2018; Netherlands	Outpatient clinic	Explore therapist behaviors; adherence; and patient outcome in digital therapy	Observational study	64; 45; NC (patients, n=45; therapists, n=19)	CBT; ND depression levels
Nakao et al [57], 2018; Japan	Outpatient medical institutions	Evaluate effectiveness of web-based bCBT in reducing therapist time in patients with depression	Single-blinded RCT	40; 20; 20; comparator: waitlist + pharmacological treatment	CBT; depression symptoms
Romijn et al [41], 2021 and Romijn et al [58];^b^ Netherlands	Outpatient specialized mental health care centers	Explore therapist fidelity to bCBT protocols for anxiety disorders	Mixed methods (derived from a larger RCT)	114; 52; 62; comparator: CBT F2F	CBT; anxiety symptoms
Tarp et al [59], 2022; Denmark	Public municipal outpatient alcohol clinics	Describe development and testing of a digital program; participant experiences; and usability of BT	Feasibility and pilot study	32; 22; NC (development: 7 therapists+3 patients)	CBT + motivational interviewing; —^s^ (intervention system usability)
Thase et al [60], 2018; United States	Department of psychiatry of medical schools	Evaluate the efficacy of computer and therapist-assisted CBT vs standard CBT	Noninferiority RCT	154; 77; 77; comparator: CBT F2F	CBT; depression symptom severity
van de Wal et al [61], 2017; Netherlands	Cancer hospitals: academic, regional, and outpatient	Investigate the efficacy of BT for FCR^t^ in cancer survivors	RCT: 2-arm, parallel group, longitudinal	88; 45; 43; comparator: TAU (any)	CBT; FCR severity
Vernmark et al [62], 2019; Sweden	Mental health care centers	Explore patient- and therapist-rated WA in bCBT and WA as a predictor for change	Exploratory secondary study from RCTs	151; 75; NC	CBT; depression levels
Witlox et al [63], 2021; Netherlands	Mental health service at general practices	Examine the effectiveness of blended ACT for older adults with anxiety	RCT (single-blinded)	314; 150; 164; comparator: TAU (FTF CBT)	ACT; anxiety severity
Wu et al [64], 2021; United States	Employer mental health program clinical services	Evaluate the outcomes of a blended care coaching program for anxiety and depression	Retrospective cohort analysis	1496; 1496; NC	CBT-based + CBT, DBT, and ACT; anxiety and depression symptoms
Wu et al [65], 2021; United States	Employer mental health program clinical services	Examine the effectiveness and the impact of bCBT on anxiety and depression	Retrospective cohort analysis	3401; 3401; NC	CBT+DBT and ACT; anxiety and depression symptoms

^a^BT: blended therapy.

^b^Linked study.

^c^WA: working alliance.

^d^RCT: randomized controlled trial.

^e^F2F: face to face.

^f^CBT: cognitive behavioral therapy.

^g^TAU: treatment as usual.

^h^iCBT-TF: internet-guided cognitive behavioral therapy with trauma focus.

^i^PTSD: posttraumatic stress disorder.

^j^NC: no comparator.

^k^iCBT: internet-delivered cognitive behavioral therapy.

^l^ND: not disclosed.

^m^PST: problem-solving therapy.

^n^bCBT: blended cognitive behavioral therapy.

^o^UTP: unified transdiagnostic protocol.

^p^ACT: acceptance and commitment therapy.

^q^DBT: dialectical behavioral therapy.

^r^BA: behavioral activation.

^s^Not applicable.

^t^FCR: fear of cancer recurrence.

Primary outcomes of individual studies included efficacy or effectiveness (14/29, 48%) [37-39,45,46,48,54,55,57, 60,61,63-65]; working alliance (3/29, 10%) [43,53,62]; usability and uptake (3/29, 10%) [50,51,59]; feasibility (2/29, 7%) [23,42]; and patient or therapist (4/29, 14%) [40,41,49,56] experience. One (4%) study [47] explored multiple primary outcomes of therapeutic alliance, compliance, and symptom reduction, and 2 (7%) studies [26,52] reported dual outcomes of efficacy and cost.

**Table 2:** Patient participants’ characteristics.

Study	Age (y), mean (SD); range	Female, n (%)	Pre-post time points, assessment tool, psychological symptoms per group: mean (SD) and severity at baseline
Askjer and Mathiasen [43], 2021	35 (13.96); 18-71	56 (74)	Baseline and 12 wk PHQ-9^a^, Depression BT^b^: 14.4 (4.1), moderate to severe Comparator: 16.05 (3.8), moderate to severe
Berger et al [45], 2018	43 (12.0); 19-73	65 (66)	Baseline and 12 wk BDI-II^c^, Depression BT: 29.6 (8.4), severe Comparator: 30.2 (11.2), severe GAD-7^d^, Anxiety BT: 11.7 (4.9), moderate Comparator: 12 (4.6), moderate
Bisson et al [46], 2022	36 (13.4); 18 to >65	125 (64)	Baseline and 16 wk CAPS-5^e^, PTSD^f^ BT: 34.6 (6.8), mild to moderate Comparator: 35.6 (6.7), mild-moderate PHQ-9, Depression BT: 15.1 (6.7), moderate to severe Comparator: 13.4 (4.6), moderate GAD-7, Anxiety BT: 13.9 (4.9), moderate Comparator: 13.4 (4.6), moderate
Cloitre et al [47], 2022	44 (11.73); 22-77	122 (60)	Baseline; 10 wk PCL-5^g^, PTSD BT: 50.7 (15.5), severe PHQ-9, Depression BT: 15.6 (5.4), moderate to severe Comparator:—^h^
Duffy et al [48], 2020	41 (13.1); 17-80	85 (69)	Baseline; at iCBT^i^ end PHQ-9, Depression BT: 15.6 (5.5), moderate to severe GAD-7, Anxiety BT: 14.8 (4.5), moderate to severe Comparator: —
Etzelmueller et al [49], 2018	55 (—); 24-64	18 (72)	Weekly assessments, — QIDS-16-SR^j^, Depression BT: 14.8 (4.4), moderate Comparator: —
Høifødt et al [37], 2013	36 (11.3); 19-63	77 (73)	Baseline and 7 wk BDI-II, Depression BT: 21.1 (6.85), moderate Comparator: 22.3 (6.7), moderate BAI^k^, Anxiety BT: 12.05 (11.1), mild Comparator: 15.3 (10.9), mild
Jacmon et al [42], 2009	35 (10.37); —	4 (44)	Baseline; 6 wk BDI-II, Depression BT: 26.5 (1.5), moderate Comparator: —
Kemmeren et al [50], 2019	42 (12.9); 18-74	129 (64)	Baseline; — PHQ-9, Depression BT: 16.2 (4.7), moderate to severe Comparator: —
Kenter et al [51], 2013	37 (10.8); 18-61	73 (70)	Baseline; 5 wk BDI-II, Depression BT: 23.5 (7), moderate Comparator: 22.4 (8.7) HADS^l^, Anxiety BT: 10.6 (2.9), moderate Comparator: 11.1 (2.4) MBI^m^, Burnout MBI-EE, BT: 2.5 (1.7); Comparator: 2.6 (1.4) MBI-D, BT: 2.0 (1.5); Comparator: 2.0 (1.5) MBI-C, BT: 3.5 (1.3); Comparator: 3.6 (1.3)
Kenter et al [52], 2015	47 (18.7); 18-91	2442 (55)	First and last F2F sessions GAF^n^, Depression group BT: 54.6 (4.9), moderate Comparator: 54.7 (4.7), moderate GAF, Anxiety group BT: 59 (5.3), moderate Comparator: 59.1 (5.2), moderate
Kok et al [38], 2014	35 (11.7); —	130 (61)	Baseline; 5 wk FQ^o^, Phobia BT: 42.4 (23.4) Comparator: 38.2 (21.9) CES-D^p^, Depression BT: 25 (8.6), severe Comparator: 24.7 (8.4), severe BAI, Anxiety BT: 45 (13.8), severe Comparator: 44.48 (13.1), severe
Kooistra et al [23], 2016	38 (8.36); 27-50	5 (55)	Baseline; 10 wk IDS-SR30^q^, Depression: BT: 40.4 (12.9), moderate BAI, Anxiety BT: 22 (12.4), moderate Comparator: —
Kooistra et al [26], 2019	39 (10.9); —	64 (63)	Baseline; 10 wk IDS-SR30, Depression: BT: 45.2 (12.1), moderate Comparator: 41.5 (11.6), moderate
Kooistra et al [53], 2020	38 (11.0); —	43 (60)	Baseline; 10 wk QIDS-SR, Depression BT: 16.6 (4.9), severe Comparator: 15.9 (4.1), severe
Lungu et al [54], 2020	33 (8.0); —	244 (64)	Baseline and 6 wk PHQ-9, Depression BT: 10.8 (4.7), moderate GAD-7, Anxiety BT: 11.7 (3.9), moderate Comparator: —
Ly et al [55], 2015	31 (11.4); 18-73	65 (70)	Baseline; 9 wk BDI-II, Depression BT: 29 (8.1), severe; Comparator: 27.3 (7.9), severe PHQ-9, Depression BT: 15.4 (4.7), moderate to severe; Comparator: 15.3 (4.5), moderate to severe BAI, Anxiety BT: 15.7 (12.1), mild to moderate; Comparator: 17.5 (9.2), moderate
Månsson et al [39], 2013	43 (15); 22-70	10 (67)	Baseline; 9 wk BAI, Anxiety BT: 18.1 (7.7), moderate GAD-7, Anxiety BT: 11.9 (6), moderate PHQ-9, Depression BT: 12.1 (6), moderate MADRS-S^r^, Depression BT: 21.2 (4), moderate Comparator: —
Månsson et al [40], 2017	30 (10.6); 18-60	36 (80)	Baseline; 12 wk PHQ-9, Depression BT: 13.3 (5.2) MADRS-S, Depression BT: 21 (8.7), moderate BAI, Anxiety BT: 20.5 (9.8) GAD-7, Anxiety BT: 10.3 (6), moderate Comparator: —
Mol et al [56], 2018	36 (12.3); 21-64	33 (73)	Baseline; approximately 26 wk QIDS, Depression BT: 15.8 (3.8), moderate to severe Comparator: —
Nakao et al [57], 2018	40 (9.7); —	20 (50)	Baseline; 12 wk GRID-HAMD^s^, Depression BT: 18.3 (3.7), moderate to severe; Comparator: 18.5 (3.6), moderate to severe BDI-II, Depression BT: 28 (8.8), moderate; Comparator: 24.4 (7.8), moderate QIDS, Depression BT: 14.8 (4.2), moderate; Comparator: 13.5 (4), moderate
Romijn et al [41], 2021	37 (11.0); 19-62	23 (52)	Baseline; 15 wk BDI-II, Depression BT: 24 (12.2), moderate; Comparator: 24 (10.3), moderate BAI, Anxiety BT: 27.9 (12), severe; Comparator: 27.15 (11.7), severe
Tarp et al [59], 2022	47 (12); 28-73	7 (32)	Addictive disorder: —
Thase et al [60], 2018	46 (14.3); —	102 (66)	Baseline and week 16 Depression: HDRS^t^ BT: 19.8 (3.5), moderate to severe Comparator: 19.6 (3.8), moderate to severe
van de Wal et al [61], 2017	59 (11.3); 31-77	47 (53)	Baseline and 12 wk CWS^u^, FCR BT: 19.6 (3.7), high; Comparator: 19.6 (3.7), high HADS-D, Depression BT: 5.9 (4.2), low; Comparator: 6.8 (4.7), low HADS-A, Anxiety BT: 8.1 (4.1), mild; Comparator: 8.4 (4.9), mild
Vernmark et al [62], 2019	35 (13.9); —	54 (74)	Baseline; 10 wk PHQ-9, Depression BT: 14.3 (5.1), moderate Comparator: —
Witlox et al [63], 2021	63 (5.70); 55-75	192 (61)	Baseline; 12 wk PHQ-9, Depression BT: 7 (4), mild; Comparator: 7.9 (3.5) GAD-7, Anxiety BT: 8.2 (4), mild to moderate; Comparator: 8.8 (3)
Wu et al [64], 2021	33 (8.62); —	921 (62)	Wk 0-7 and wk 8-15 PHQ-9, Depression BT: 6 (3), mild GAD-7, Anxiety BT: 9.6 (2), mild to moderate Comparator: —
Wu et al [65], 2021	33 (8.68); —	2218 (65)	Wk 0-7 and wk 8-15 PHQ-9, Depression BT: 10.7 (4.9), moderate GAD-7, Anxiety BT: 11.8 (4.1), moderate Comparator: —

^a^PHQ-9: Patient Health Questionnaire-9.

^b^BT: blended therapy.

^c^BDI-II: Beck Depression Inventory II.

^d^GAD-7: Generalized Anxiety Disorder-7.

^e^CAPS-5: Clinician-Administered Posttraumatic Stress Disorder Scale for the Diagnostic and Statistical Manual of Mental Disorders, Fifth Edition.

^f^PTSD: posttraumatic stress disorder.

^g^PCL-5: Posttraumatic Stress Disorder Checklist for the Diagnostic and Statistical Manual of Mental Disorders, Fifth Edition.

^h^Not available.

^i^iCBT: internet-delivered cognitive behavioral therapy.

^j^QIDS-16-SR: 16-Item Quick Inventory of Depressive Symptomatology, self-reported.

^k^BAI: Beck Anxiety Inventory.

^l^HADS: Hospital Anxiety and Depression Scale.

^m^MBI: Maslach Burnout Inventory.

^n^GAF: Global Assessment of Functioning.

^o^FQ: Fear Questionnaire.

^p^CES-D: Centre for Epidemiologic Studies Depression Scale.

^q^IDS-SR30: Inventory of Depressive Symptomatology Self-Rated.

^r^MADRS-S: Montgomery Åsberg Depression Rating Scale—self-rating version.

^s^GRID-HAMD: 17-item GRID-Hamilton Depression Rating Scale score.

^t^HDRS: Hamilton Depression Rating Scale.

^u^CWS: Cancer Worry Scale.

### Classification of BT Models

On the basis of the models defined by Erbe et al [24], the majority (26/29, 90%) of the studies reported an *integrated* intervention design, where new therapeutic content was delivered either (1) across both the face-to-face and digital modalities (*core*; n=14) or (2) primarily using one modality (usually the face-to-face component) with additional content delivered as *supplementary material* (n=12). Integrated interventions were the most common designs for addressing depression (n=14) and depression and or anxiety (n=8). Three studies were classified as *sequential* designs and described a *core* role for both face-to-face and digital components. One sequential model was delivered as *stepped care* in which most patients received digital therapy only.

BT delivery was further differentiated based on whether both components were delivered in a preset, *alternate* format or a tailored, *case-by-case* arrangement. Most integrated interventions (17/26, 65%) used an *alternate* delivery format. Therapeutic content within integrated and alternate designs had either *core* (9/17, 53%) or *supplementary* (8/17, 47%) roles.

In 19 (65%) studies, patients engaged with digital content following a *standardized* program with minimal tailoring of the materials presented. In contrast, 10 (35%) studies described a more *personalized* manner of assigning or interacting with digital content where the therapist and patient have more autonomy to choose, change, or create digital content according to individual need. An overview of model classifications is presented in Table 3.

**Table 3:** Blended therapy model classification per study.

Study	Interaction of F2F^a^ and digital components			Role of F2F and digital components			Pattern of delivery of F2F and digital			Digital content delivery	
	Integrated	Sequential	Core		Supplementary	Alternate		Case by case	Personalized		Standardized
Askjer and Mathiasen [43], 2021	✓		✓			✓					✓
Berger et al [45], 2018	✓				✓			✓	✓		
Bisson et al [46], 2022	✓				✓			✓	✓		
Cloitre et al [47], 2022	✓				✓	✓					✓
Duffy et al [48], 2020^b^		✓	✓						✓		
Etzelmueller et al [49], 2018	✓				✓			✓	✓		
Høifødt et al [37], 2013	✓		✓			✓					✓
Jacmon et al [42], 2009	✓		✓					✓			✓
Kemmeren et al [50], 2019	✓		✓			✓					✓
Kenter et al [51], 2013^b^		✓	✓								✓
Kenter et al [52], 2015	✓		✓					✓			✓
Kok et al [38], 2014^b^		✓	✓								✓
Kooistra et al [23], 2016	✓				✓	✓					✓
Kooistra et al [26], 2019	✓				✓	✓					✓
Kooistra et al [53], 2020	✓				✓	✓					✓
Lungu et al [54], 2020	✓				✓	✓			✓		
Ly et al [55], 2015	✓				✓	✓			✓		
Månsson et al [39], 2013	✓				✓			✓	✓		
Månsson et al [40], 2017	✓				✓			✓	✓		
Mol et al [56], 2018	✓		✓			✓					✓
Nakao et al [57], 2018	✓		✓			✓			✓		
Romijn G et al [41], 2021	✓		✓			✓					✓
Tarp et al [59], 2022	✓		✓					✓			✓
Thase et al [60], 2018	✓		✓			✓					✓
van de Wal et al [61], 2017	✓				✓	✓					✓
Vernmark et al [62], 2019	✓		✓			✓					✓
Witlox et al [63], 2021	✓		✓			✓					✓
Wu et al [64], 2021	✓		✓					✓	✓		
Wu et al [65], 2021	✓		✓					✓	✓		

^a^F2F: face to face.

^b^Sequential models present a *linear* pattern of delivery.

### Structure, Content, and Ratio of Sessions in BT Models

#### Overview

Within the *integrated* model, 81% (21/26) of studies reported commencing BT with the face-to-face component. Five *integrated* interventions began treatment with the digital component; of those, 4 [38,42,47,51] used the digital component as the intervention “anchor”—that is, the digital component led the therapeutic process. One [48] *sequential* intervention (stepped care) used the digital modality as a prequel for high-intensity face-to-face treatment based on patient symptom severity. Digital sessions were mostly delivered via website platforms with individualized access. Overall, digital session components presented CBT-based content and followed the frameworks used in face-to-face settings. Digital content was typically asynchronous. Intervention structure and content is summarized in Multimedia Appendix 4.

#### Face-to-Face Versus Digital Sessions Distribution in BT Models

In total, 26 (87%) studies reported the number of face-to-face sessions, which ranged from 3 to 21 sessions (mean 7, SD 4.2). Of those reporting the number of face-to-face sessions, 24 (92%) used an *integrated* intervention design. Mean face-to-face session duration across studies was 49 minutes (SD 11.7, range 27-65 min/session). Periodicity of sessions were reported by 24 studies, with most of those studies describing face-to-face sessions as delivered weekly (12/24, 50%) [23,26,37,39,41,42,45,49,53,57,59,64].

A total of 20 (69%) studies reported on the number of digital sessions (mean 8, SD 3, range 4-14 sessions)—of those studies, 18 (90%) presented an *integrated* design. Although the mean time for digital sessions was largely undefined and unreported, 12 (41%) studies [37,40,45,47,50,52,57,60-63,65] reported that digital modules were typically developed to range between 15 and 60 minutes. Eight (28%) studies [42,45,46,48,57,59,60,64] allowed patients to complete modules at their own pace; 13 (45%) studies [39,40,42,45,48,49,54-56,60,61,64,65] reported digital sessions’ periodicity to be flexible; weekly (14/29, 48%) [23,26,37,38,41,46,47,51-53,57,59,62,63] or fortnightly (2/29, 7%) [43,50] completion was also reported.

#### Face-to-Face and Digital Ratio

In total, 16 (55%) studies [23,26,37,41,42,45-50,53,56,60,62,65] reported the ratio of face-to-face and digital sessions. Most of those studies had an *integrated* design (14/16, 88%) and typically delivered sessions at a 1:1 ratio (1 face-to-face to 1 digital).

#### Treatment Length

The BT mean length of treatment was 12 (SD 5.1, range 6-26) weeks; however, this was unreported for 5 studies [38,49,51,52,63] (Table 4).

**Table 4:** Model classification versus session structure.

		Integrated (n=26)	Sequential (n=3)	Core (n=17)	Supplementary (n=12)	Alternate (n=17)	Case by case (n=9)	Linear^a^ (n=3)	Personalized (n=10)	Standardized (n=19)
Treatment length^b^, mean (range); n		12 (6-21); 23	21; (—^c^); 1	13 (6-26); 13	11 (6-21); 11	13 (6-26); 16	10 (6-12); 7	21; (—); 1	10 (6-21); 9	14 (8-26); 15
**Face-to-face sessions**										
	Number, mean (range); n	8 (3.5-21); 23	3.5 (0-10); 3	6 (0-12); 15	9 (4-21); 11	7 (3.5-12); 17	9 (4-21); 6	10; (—); 1	9 (4-21); 9	6 (0-12); 17
	Time (min), mean (range); n	49 (27-65); 17	NR^d^; 0	47 (27-65); 9	51 (45-65); 8	46.5 (27-65); 12	55 (45-60); 5	NR; 0	53 (44-60); 6	47 (27-65); 11
	Periodicity	1w^e^: n=12; 2w^f^: n=4; 3w^g^: n=1; var^h^: n=7	NR; (0)	1w: n=6; 2w: n=3; 3w: n=1; var: n=3	1w: n=6; var: n=4; 2w: n=1	1w: n=6; 2w: n=4; 3w: n=1; var: n=6	1w: n=6; var: n=1	NR	1w: n=5; var: n=3	1w: n=7; 2w: n=4; 3w: n=1; var: n=4
**Digital sessions**										
	Number, mean (range); n	9 (4-14); 17	4 (4-5); 2	7 (4-14); 11	9 (7-14); 8	7 (4-10); 11	10 (6-14); 6	4; 1	8 (4-14); 5	8 (4-14); 15
	Time (min), mean (range); n	38 (6-65); 8	NR; 0	44 (30-65); 4	32 (6-62); 4	38 (28-65); 5	38 (6-62); 3	NR; 0	36 (6-62); 4	40 (28-65); 4
	Periodicity	1w: n=13; 2w: n=1; var: n=12	2w: n=1; any^i^: n=2	1w n=9; 2w: n=2; any: n=6	2w: n=4; any: n=8	1w: n=9; 2w: n=2; any: n=6	1w: n=2; any: n=7	1w: n=2; any: n=1	1w: n=1; any: n=9	1w: n=12; 2w; n=2; any: n=5

^a^*Linear* describes the delivery pattern typical of *sequential* designs.

^b^Treatment length is measured in weeks.

^c^Not applicable.

^d^NR: not reported.

^e^1w: weekly.

^f^2w: 2-weekly.

^g^3w: 3-weekly.

^h^var: variable (eg, varied from weekly to fortnightly or other pattern).

^i^any: anytime (ie, no pattern from the outset).

### Outcomes of BT Interventions

#### Treatment Uptake

In total, 25 (86%) studies reported on BT uptake mean rates (mean 91%, SD 10.2%, range 60%-100%). Of those, 24 (96%) studies [23,26,37-43,45-48,50,51,53,55-57,59-63] reported a mean uptake of 92% (SD 8.2%, range 73%-100%) and 1 (4%) study [49] reported a lower uptake (60%). A total of 14 (48%) studies reported on treatment completion rates, with an average of 61% (SD 29.4%, range 11.5%-100%) of patients completing treatment.

#### Treatment Adherence

Our review considered adherence as completing a minimum number of sessions determined by each study. However, adherence criteria differed across studies and there was a lack of data to confirm whether session structure or ratio influenced adherence to intervention. A total of 23 (279%) studies reported on BT adherence rates, with a mean of 81% (SD 11.8%). In total, 20 (69%) *integrated* studies reported on BT adherence (mean 83%, SD 11%, range 62%-100%). In contrast, all *sequential* studies (3/29, 10%) had a comparatively lower mean adherence (64%, SD 1.8). Dropout rates were reported in 25 (86%) studies, with 24 of those reporting <40% dropout rates (mean 18.5%, SD 17.2%, range 0%-38%). The intervention with the lowest adherence rate (16%) and highest dropout rate (84%) [38] had a *sequential* design.

Regarding the role of components within BT, interventions with *supplementary* designs presented higher adherence (mean 88%, SD 9%, range 72.5%-100%) than *core* designs (mean 76%, SD 11.7%, range 16%-100%). Details on the uptake and adherence reported per study are available in Multimedia Appendix 5.

#### Health Service Outcomes

Four (14%) studies reported on the impact of BT on therapist time: 2 studies [42,55] found a decrease, one study reported an increase [52], and one found no change [26] in the time needed to deliver therapy. Findings related to efficacy and costs were mixed, with one study [52] suggesting that BT was not cost-effective compared with face-to-face therapy, while another study [55] highlighted reduced costs due to the potential of BT for treating twice as many patients as compared with face-to-face treatment.

#### Patient Satisfaction and Working Alliance

Eight (28%) studies [23,37,38,43,45,46,49,63] reported on patient satisfaction with BT treatment—criteria for determining patient satisfaction were heterogeneous, but all studies reported it as “high.” Four (14%) studies [43,47,53,62] reported on working alliance and reported it as “high.” Two (7%) studies [43,62] suggested that the therapist-rated working alliance predicted treatment outcomes and that this may be specific to BT.

#### Barriers to and Facilitators of Intervention Uptake and Engagement

Potential barriers to intervention uptake reported [23,46,49,51,59] included *a lack of understanding about the intervention* and *digital challenges*. Reported [42,51,59] facilitators to intervention uptake included *convenience and flexibility of the digital component, anonymity,* and *autonomy enabled by the BT design.* Barriers to intervention engagement identified [37,38,42,45,46,48-50,56,61] included *the good enough effect* (ie, when patients drop out during the initial stages of the intervention arguing they “feel better” and “no longer need therapy” [66]), *being left unchecked, reduced therapy support* (ie, lack of therapist follow-up regarding digital activities), and *digital challenges*. Facilitators of intervention engagement included *experience in the use of technology, a program tailored to patient-specific needs, patient-therapist digital communication between sessions,* and *a patient-therapist working alliance fostered throughout the intervention* [37-39,43,45-50,52,57,64,65]*.*

#### Treatment Efficacy

We conducted a meta-analysis to examine differences in treatment outcomes. There were only a sufficient number of studies to meta-analyze depression and anxiety outcomes.

Data pooled from 9 RCT studies demonstrated a moderate to large, significant improvement in depression symptoms (Cohen *d*=–1.1, 95% CI –0.6 to –1.6, *P*<.001). However, comparing treatment outcomes for anxiety interventions with controls (n=5), there was no significant improvement across studies (Cohen *d*=–0.1, 95% CI –0.3 to 0.05, *P*=.17). Between-study heterogeneity was high and did not change after conducting publication bias assessment using the funnel plot trim and fill method, both in the depression (*Q*=90.3; *P*<.001; *I^2^*=91.1; *T^2^*=0.1; *T*=0.7) and anxiety groups (*Q*=1.1; *P*=.17; *I^2^*=0; *T^2^*=0; *T*=0). We noted that estimates of heterogeneity are impacted by the very small number of studies analyzed (<10 studies).

Meta-analysis was also conducted to examine associations between depression and anxiety scores on various scales and BT structure. For depression, mixed-effect analysis suggested higher effects sizes for interventions where the therapeutic content was delivered primarily face to face with digital content *supplementing* the face-to-face content (n=11; Cohen *d*=–0.75, 95% CI –0.56 to –0.95) compared with digital therapeutic content delivered as *core* (n=10; Cohen *d*=–0.5, 95% CI –0.4 to –0.7)—however, differences were not statistically significant (*P*<.08). Similar associations were found for anxiety, with higher ESs for *supplementary* (n=8; Cohen *d*=–0.9, 95% CI –0.6 to –1.2) compared with *core* structure (n=5; Cohen *d*=–0.6, 95% CI –0.22 to –0.98). Mixed-effect analysis also indicated statistically significantly (*P*<.001) higher ESs for interventions with ≤6 face-to-face sessions both for depression (n=9; Cohen *d*=–0.7, 95% CI –0.5 to –0.9) and anxiety (n=7; Cohen *d*=–0.8, 95% CI –0.3 to –1.3) compared with interventions with >6 face-to-face sessions for depression (n=11; Cohen *d*=–0.6, 95% CI –0.4 to –0.8) and anxiety (n=5; Cohen *d*=–0.7, 95% CI –0.4 to –1). Similarly, interventions with >50% of sessions delivered digitally had higher ESs (*P*<.001) both for depression (n=5; Cohen *d*=–0.8, 95% CI –0.6 to –1.1) and anxiety (n=4; Cohen *d*=–0.8, 95% CI 0.006 to –1.6) compared with interventions where >50% sessions were delivered face to face, both for depression (n=9; Cohen *d*=–0.5, 95% CI –0.3 to –0.7) and anxiety (n=3; Cohen *d*=–0.58, 95% CI –0.3 to –0.9). Data regarding meta-analyses are available in Multimedia Appendix 6.

## Discussion

### Overview

We reviewed blended psychological therapy models and classified them according to their structure, content, and ratio of face-to-face and digital sessions. Most BT interventions were CBT-based and addressed depression—for which models with *integrated* and s*upplementary* designs resulted in improved treatment efficacy*.* Interventions typically used an *integrated* design with the face-to-face component “anchoring” the intervention. Essential, therapeutic content across treatment designs was typically delivered as both face to face and digital (ie, a *core* design) and in an *alternate* pattern. However, several studies used digital components to *supplement* therapeutic content delivered face to face. Most interventions also relied on *standardized* digital content rather than content tailored to individual patients.

Our study confirms that BT leads to improved overall patient uptake (mean 91%) and adherence (mean 81%), contrasting with the lower uptake and adherence rates previously reported for digital therapy alone. For example, an observational cohort study analyzed clinical data from 15,882 patients assessed for digital-only treatment of various psychological disorders, reporting 22% uptake and 68% adherence rates [14]. In addition, review studies on digital therapy for depression and anxiety symptoms [16] and for depression alone [17] indicated mean uptake rates of 56% (range 21%-88%) and 88% (range 42%-100%), respectively, as well as mean adherence rates of 18% (range 7%-42%) and 60% (range 14%-93%), respectively. These contrasting findings suggest a potential connection between improved engagement with digital components when these are integrated into an intervention with a more prominent role of the therapist, that is, in a blended format.

### Meta-Analysis Results: Treatment Versus Control Dyads

Across CBT-based RCTs, symptom reduction was observed for both depression and anxiety—although the reduction in anxiety symptoms was not substantial. Our result contrasts with overall findings on effectiveness of digital-only interventions exclusively addressing anxiety [67], which indicate effective outcomes. However, a subgroup analysis in that study confirmed similar ESs of both digital and face-to-face anxiety treatments. The lack of significance of our results might reflect the fact that only 1 included study addressed anxiety as a primary intervention outcome, while the other 4 interventions primarily targeted posttraumatic stress disorder, Fear of Cancer Recurrence, or depression. This suggests that transdiagnostic interventions may not be sufficient for treating anxiety—even with therapist guidance.

### Meta-Analysis Results: BT Treatment Outcomes Versus the BT Model

Our analysis indicated that interventions that delivered face-to-face sessions for depression and anxiety in a lower ratio (≤50%) and in a lower number (≤6) had higher, statistically significant overall ESs. As the ratio of face-to-face sessions was similar in *core* or *supplementary* models, it is unclear what role digital sessions played in the delivery of therapeutic content compared with simply supplementing face-to-face sessions. Moreover, ESs for depression and anxiety in *supplementary* designs (Cohen *d*=–0.75 and Cohen *d*=–0.87, respectively) were higher than those in *core* designs (Cohen *d*=–0.53 and Cohen *d*=–0.6, respectively). Those findings suggest that therapeutic content may achieve better results when introduced by therapists and reinforced digitally—a characteristic of *supplementary* models, in which the digital component extends or reinforces face-to-face content. In addition, s*upplementary* models might provide a more seamless transition between face-to-face and digital content as therapists can identify and discuss challenging topics with patients before they go on the web, what could result in enhanced patient engagement, adherence and treatment results. This argument is supported by studies on participants’ preference for BT models that enable greater therapist-patient interaction [21,50,68,69]. In addition, participants’ views reported across our study also suggest that improved digital access and support from therapist facilitated BT engagement.

The higher ESs of *supplementary* versus *core* designs may also reflect the therapists’ preference for face-to-face contact. This argument finds support in studies on health professionals’ preferences regarding face-to-face versus digital delivery, both in blended [68,70] and digital-only [20,71] interventions. Those studies suggest that, despite recognizing the advantages of digital interventions, professionals perceive face-to-face delivery as more attractive than digital delivery, which could influence the endorsement of digital therapy delivery as a supplement. This suggests that therapists might feel more comfortable *engaging with* and *promoting* the digital arm in a *supplementary* way.

In addition, it is possible that both therapists and patients expect the “bulk” of the therapeutic content to be delivered face to face in blended interventions, explaining why we found BT *core* models to be less efficacious than *supplementary*. Therapy expectations would also help explain the contrast of our results with research [67,72] on using digital therapy alone for depression and anxiety, which found digital interventions to be as good as or better than face-to-face interventions. Perhaps because patients engaging in digital-only treatment would *expect* therapeutic content to be delivered digitally, as there is no face-to-face option, they fare better on the digital component than they would in a BT model.

### Optimizing Effectiveness, Time, and Resources in BT Treatment

One aim of BT interventions [24] is to enable better balance between treatment effects; patient engagement; treatment time; and the use of resources for both patients and therapists. BT models with *integrated, core, alternate,* and *standardized* designs allow for optimized treatment delivery. However, the results of our meta-analysis suggest that *supplementary* BT models are more effective. Considering these arguments, perhaps a midway alternative would be to promote a more digitally focused role of the therapist in a blended design. Following this idea, therapists would support and encourage patients to complete digital content as an enhancing adjunct to face-to-face contact—and not only as an “add-on” feature. A more digitally focused role of the therapist might promote increased patient engagement, enabling improved outcomes with lower doses of face-to-face treatment.

### Study Limitations

We optimized database search terms with the assistance of a librarian; however, it is possible that eligible studies were not included in this review. In addition, the analysis did not assess the potential impact of heterogeneities found among selected studies on the variables analyzed, for example, whether different populations, settings, or therapeutic approach applied in the interventions might affect uptake or adherence or even the way blended sessions are delivered. Furthermore, the small number of studies included in our meta-analysis impacted the breadth of our results. In addition, there were limited studies describing intervention structure and content of both face-to-face and digital components in detail. Despite having contacted authors regarding missing data, several gaps remained on key variables investigated (eg, number and time of face-to-face and digital sessions, adherence parameters, and acceptability of intervention), which impacted the depth of analysis. Hence, the outcomes reported in this study should be interpreted with caution.

### Recommendations

Despite growing evidence regarding BT efficacy, the lack of clearer, detailed data reporting on its structure and content poses challenges to scientific reproducibility of BT, possibly affecting its implementation success. Details related to the number, time, and distribution of sessions; specific content of both digital and face-to-face sessions, as well as the role, relevance, and influence of the digital component within the therapy plan; feedback on face-to-face and digital session content; and the use of assigned digital materials as well as the perception of its usefulness from the perspective of both therapists and patients are examples of useful data that are not adequately reported. We recommend that future studies include a more detailed reporting of methodology, particularly regarding the structure and content of sessions.

### Conclusions

This systematic review examined blended models for the treatment of psychological disorders to identify what aspects of BT underpin effective treatment and improved engagement. Evidence suggests that implementing an integrated model is feasible in the treatment of psychological disorders. BT was reported as being either more effective or noninferior to face-to-face treatment, particularly when applied to the treatment of anxiety and depression. BT interventions studied reported high mean uptake and adherence rates, showing promise in improving engagement to treatment. Higher ESs were found for depression and anxiety outcomes in interventions with *integrated*, *supplementary* models; with a lower number of face-to-face sessions; and with a lower ratio of face-to-face versus digital sessions, suggesting that combining a more digitally focused therapist role with fewer face-to-face sessions can be effective and increase access to treatment.

To support improved reporting, we have developed a taxonomy for BT models based on the key themes identified in this review regarding model structure and components. Future studies detailing the structure and content of BT models may help identify suitable models for the treatment of different psychological disorders.

## Appendix

Multimedia Appendix 1 PRISMA (Preferred Reporting Items for Systematic Reviews and Meta-Analyses) checklist.

Multimedia Appendix 2 Database searches: examples.

Multimedia Appendix 3 Quality assessment.

Multimedia Appendix 4 Blended therapy structure: content described.

Multimedia Appendix 5 Uptake: adherence described.

Multimedia Appendix 6 Meta-analyses data: graphs.

## Abbreviations

BT: blended therapy
CBT: cognitive behavioral therapy
ES: effect size
PRISMA: Preferred Reporting Items for Systematic Reviews and Meta-Analyses
RCT: randomized controlled trial
