# Supplementary material for: Blended Psychological Therapy for the Treatment of Psychological Disorders in Adult Patients: Systematic Review and Meta-Analysis
Source: Interact J Med Res. 2024 Oct 29;13:e49660. doi: 10.2196/49660 (PMC11558224; doi:10.2196/49660)
Supplement: Multimedia Appendix 2 [file ijmr_v13i1e49660_app2.pdf]

Database searches examples

CINAHL

‹ Back

EBSCOhost

Folder List

Name of Search/Alert	CINAHL 2022-2023
Description	feb 23
Date Created	2/17/2023
Databases	[illegible]<br>RILM Abstracts of Music Literature with Full Text<br>MasterFILE Reference eBook Collection<br>Vanity Fair Magazine Archive<br>CINAHL Complete
Search Strategy	blended OR (online and face to face)
Interface	EBSCOhost
Save Search As	◉ Saved Search (Permanent)<br>○ Saved Search (Temporary, 24 hours)<br>○ Alert

Save Cancel

Search History

#	Query	Limiters/Expanders	Last Run Via
S1	blended OR (online and face to face)	Limiters - Published Date: 20220501-20230231; English Language; Age Groups: Adult: 19-44 years, Middle Aged: 45-64 years, Aged: 65+ years, Aged, 80 and over, All Adult<br>Expanders - Apply equivalent subjects<br>Search modes - Boolean/Phrase	Interface - EBSCOhost Research Databases<br>Search Screen - Advanced Search<br>Database - CINAHL Complete

Save Cancel

Top of Page

Download on the App Store | GET IT ON Google Play | EBSCO Connect | Privacy Policy | A/B Testing | Terms of Use | Copyright | Cookie Policy | Manage

© 2023 EBSCO Industries, Inc. All rights reserved.

EMBASE

Set	Search Statement
1.	(Online or Internet or Web-based or Computer or e-therapy or Digital).mp. [mp=title, abstract, heading word, table of contents, key concepts, original title, tests & measures, mesh word]
2.	limit 1 to english language
3.	blended.mp. [mp=title, abstract, heading word, table of contents, key concepts, original title, tests & measures, mesh word]
4.	limit 3 to english language
5.	(treatment or therapy or care).mp. [mp=title, abstract, heading word, table of contents, key concepts, original title, tests & measures, mesh word]
6.	limit 5 to english language
7.	("psychological distress" or "psychological disease").mp. [mp=title, abstract, heading word, table of contents, key concepts, original title, tests & measures, mesh word]
8.	limit 7 to english language
9.	2 and 6 and 8
10.	4 and 6 and 8
11.	9 or 10
12.	limit 11 to (english language and english and (adult <18 to 64 years> or aged <65+ years>) and "humans only (removes records about animals)")
13.	limit 12 to yr="2021 -Current"
14.	limit 13 to yr="2022 -Current"

MEDLINE

Set	Search Statement
1.	(Online or Internet or Web-based or Computer or e-therapy or Digital).mp. [mp=title, abstract, heading word, table of contents, key concepts, original title, tests & measures, mesh word]
2.	limit 1 to english language
3.	blended.mp. [mp=title, abstract, heading word, table of contents, key concepts, original title, tests & measures, mesh word]
4.	limit 3 to english language
5.	(treatment or therapy or care).mp. [mp=title, abstract, heading word, table of contents, key concepts, original title, tests & measures, mesh word]
6.	limit 5 to english language
7.	psychological.mp. [mp=title, abstract, heading word, table of contents, key concepts, original title, tests & measures, mesh word]
8.	limit 7 to english language
9.	(distress or disease).mp. [mp=title, abstract, heading word, table of contents, key concepts, original title, tests & measures, mesh word]
10.	limit 9 to english language
11.	2 and 6 and 8 and 10
12.	4 and 6 and 8 and 10
13.	11 or 12
14.	limit 13 to (english language and ("young adult (19 to 24 years)" or "adult (19 to 44 years)" or "young adult and adult (19-24 and 19-44)" or "middle age (45 to 64 years)" or "middle aged (45 plus years)" or "all aged (65 and over)" or "aged (80 and over)") and english and (adaptive clinical trial or classical article or clinical study or clinical trial, all or clinical trial, phase i or clinical trial, phase ii or clinical trial, phase iii or clinical trial, phase iv or clinical trial protocol or clinical trial protocols as topic or clinical trial or comparative study or controlled clinical trial or dataset or english abstract or equivalence trial or evaluation study or government publication or guideline or journal article or meta analysis or multicenter study or observational study or practice guideline or pragmatic clinical trial or randomized controlled trial or research support, nih, extramural or research support, nih, intramural or "review" or "scientific integrity review" or "systematic review" or technical report or twin study or validation study))
15.	limit 14 to yr="2021 -Current"
16.	limit 15 to yr="2022 -Current"

PROQUEST

☐ 10	Name:	PROQUEST May22-Mar23 Edit name
	Searched for:	noft(blended OR (online AND "face to face")) AND noft(disorder OR distress) AND psycholog* AND la.exact("English") AND pd(>20220501)
	Limited by:	Date: After 01 May 2022<br>Language: English
	Databases:	ProQuest Central
	Notes:	Add notes
	Saved:	17 February 2023

Modify Search   Delete   Create alert   Create RSS feed   Get link

PSYCHINFO

Set	Search Statement
1.	(Online or Internet or Web-based or Computer or e-therapy or Digital).mp. [mp=title, abstract, heading word, table of contents, key concepts, original title, tests & measures, mesh word]
2.	limit 1 to english language
3.	blended.mp. [mp=title, abstract, heading word, table of contents, key concepts, original title, tests & measures, mesh word]
4.	limit 3 to english language
5.	(treatment or therapy or care).mp. [mp=title, abstract, heading word, table of contents, key concepts, original title, tests & measures, mesh word]
6.	limit 5 to english language
7.	psychological.mp. [mp=title, abstract, heading word, table of contents, key concepts, original title, tests & measures, mesh word]
8.	limit 7 to english language
9.	(distress or disease).mp. [mp=title, abstract, heading word, table of contents, key concepts, original title, tests & measures, mesh word]
10.	limit 9 to english language
11.	2 and 6 and 8 and 10
12.	4 and 6
13.	11 or 12
14.	limit 13 to yr="2021 -Current"
15.	limit 14 to yr="2022 -Current"
