# Supplementary material for: Blended Psychological Therapy for the Treatment of Psychological Disorders in Adult Patients: Systematic Review and Meta-Analysis
Source: Interact J Med Res. 2024 Oct 29;13:e49660. doi: 10.2196/49660 (PMC11558224; doi:10.2196/49660)
Supplement: Multimedia Appendix 3 [file ijmr_v13i1e49660_app3.pdf]

Quality Assessment*

1. Controlled studies: NIH Quality Assessment of Controlled Intervention Studies[1]

Source: Study Quality Assessment Tools - https://www.nhlbi.nih.gov/health-topics/study-quality-assessment-tools

Quality Assessment – Questions:

1. Was the study described as randomized, a randomized trial, a randomized clinical trial, or an RCT?
2. Was the method of randomization adequate (i.e., use of randomly generated assignment)?
3. Was the treatment allocation concealed (so that assignments could not be predicted)?
4. Were study participants and providers blinded to treatment group assignment?
5. Were the people assessing the outcomes blinded to the participants' group assignments?
6. Were the groups similar at baseline on important characteristics that could affect outcomes (e.g., demographics, risk factors, co-morbid conditions)?
7. Was the overall drop-out rate from the study at endpoint 20% or lower of the number allocated to treatment?
8. Was the differential drop-out rate (between treatment groups) at endpoint 15 percentage points or lower?
9. Was there high adherence to the intervention protocols for each treatment group?
10. Were other interventions avoided or similar in the groups (e.g., similar background treatments)?
11. Were outcomes assessed using valid and reliable measures, implemented consistently across all study participants?
12. Did the authors report that the sample size was sufficiently large to be able to detect a difference in the main outcome between groups with at least 80% power?
13. Were outcomes reported or subgroups analysed prespecified (i.e., identified before analyses were conducted)?
14. Were all randomized participants analysed in the group to which they were originally assigned, i.e., did they use an intention-to-treat analysis?

Papers assessed:	*Average quality rating:*
Askjer et al 2021	good
Berger et al, 2018	good
Bisson et al, 2022	good
Høifødt et al, 2013	fair
Kok et al., 2014	fair
Kooistra et al 2019	good
Kooistra et al 2020	good
Ly et al, 2015	good
Nakao et al, 2018	good
Thase et al. 2018	good
van de Wal et al 2017	good
Witlox et al, 2021	good

2. Pre-post with no control studies: NIH Quality Assessment Tool for Before-After (Pre-Post) Studies With No Control Group[1]

Source: Study Quality Assessment Tools - https://www.nhlbi.nih.gov/health-topics/study-quality-assessment-tools

Quality Assessment – Questions:

1. Was the study question or objective clearly stated?
2. Were eligibility/selection criteria for the study population prespecified and clearly described?
3. Were the participants in the study representative of those who would be eligible for the test/service/intervention in the general or clinical population of interest?
4. Were all eligible participants that met the prespecified entry criteria enrolled?
5. Was the sample size sufficiently large to provide confidence in the findings?
6. Was the test/service/intervention clearly described and delivered consistently across the study population?
7. Were the outcome measures prespecified, clearly defined, valid, reliable, and assessed consistently across all study participants?
8. Were the people assessing the outcomes blinded to the participants' exposures/interventions?
9. Was the loss to follow-up after baseline 20% or less? Were those lost to follow-up accounted for in the analysis?
10. Did the statistical methods examine changes in outcome measures from before to after the intervention? Were statistical tests done that provided p values for the pre-to-post changes?
11. Were outcome measures of interest taken multiple times before the intervention and multiple times after the intervention (i.e., did they use an interrupted time-series design)?
12. If the intervention was conducted at a group level (e.g., a whole hospital, a community, etc.) did the statistical analysis take into account the use of individual-level data to determine effects at the group level?

Papers assessed:	*Average quality rating:*
Jacmon et al, 2009	fair
Kemmeren et al, 2019	good
Kooistra et al 2016	fair
Månsson et al, 2013	fair
Månsson et al, 2017	fair
Romijn G, et al., 2021	fair
Vernmark et al, 2019	good

3. Cohort studies: NIH Quality Assessment Tool for Observational Cohort and Cross-Sectional Studies[1]

Source: Study Quality Assessment Tools - https://www.nhlbi.nih.gov/health-topics/study-quality-assessment-tools

Quality Assessment – Questions:

1. Was the research question or objective in this paper clearly stated?
2. Was the study population clearly specified and defined?
3. Was the participation rate of eligible persons at least 50%?
4. Were all the subjects selected or recruited from the same or similar populations (including the same time period)? Were inclusion and exclusion criteria for being in the study prespecified and applied uniformly to all participants?
5. Was a sample size justification, power description, or variance and effect estimates provided?
6. For the analyses in this paper, were the exposure(s) of interest measured prior to the outcome(s) being measured?
7. Was the timeframe sufficient so that one could reasonably expect to see an association between exposure and outcome if it existed?
8. For exposures that can vary in amount or level, did the study examine different levels of the exposure as related to the outcome (e.g., categories of exposure, or exposure measured as continuous variable)?
9. Were the exposure measures (independent variables) clearly defined, valid, reliable, and implemented consistently across all study participants?
10. Was the exposure(s) assessed more than once over time?
11. Were the outcome measures (dependent variables) clearly defined, valid, reliable, and implemented consistently across all study participants?
12. Were the outcome assessors blinded to the exposure status of participants?
13. Was loss to follow-up after baseline 20% or less?
14. Were key potential confounding variables measured and adjusted statistically for their impact on the relationship between exposure(s) and outcome(s)?

Papers assessed:	*Average quality rating:*
Cloitre et al, 2022	good
Duffy et al, 2020	good
Kenter et al. 2013	good
Kenter et al. 2015	good
Lungu et al, 2020	good
Mol et al, 2018	good
Wu, Chen et al, 2021	good
Wu et al, 2021	good

4. Qualitative study: CASP Checklist[2]

Source: CASP Checklist: 10 questions to help you make sense of a Qualitative research - https://casp-uk.net/casp-tools-checklists/

Quality Assessment – Questions:

1. Was there a clear statement of the aims of the research?
2. Is a qualitative methodology appropriate?
3. Was the research design appropriate to address the aims of the research?
4. Was the recruitment strategy appropriate to the aims of the research?
5. Was the data collected in a way that addressed the research issue?
6. Has the relationship between researcher and participants been adequately considered?
7. Have ethical issues been taken into consideration?
8. Was the data analysis sufficiently rigorous?
9. Is there a clear statement of findings?
10. How valuable is the research?

Paper assessed:	*Average quality rating:*
Etzelmueller et al, 2018	good

5. Mixed-method study: Mixed Methods Appraisal Tool (MMAT), version 2018[3]

Source: Part I: MMAT, version 2018 - https://www.nccmt.ca/knowledge-repositories/search/232%20(accessed%20May%202017)

Quality Assessment – Questions:

1. Is there an adequate rationale for using a mixed methods design to address the research question?
2. Are the different components of the study effectively integrated to answer the research question?
3. Are the outputs of the integration of qualitative and quantitative components adequately interpreted?
4. Are divergences and inconsistencies between quantitative and qualitative results adequately addressed?
5. Do the different components of the study adhere to the quality criteria of each tradition of the methods involved?

Paper assessed:	*Average quality rating:*
Tarp et al, 2022	good

***Paper quality rating conducted by researchers Kelly Nunes-Zlotkowski & Joanne Shaw**

References:

1. NIH National Heart L, and Blood Institute. Study quality assessment tools. U.S. Department of Health and Human Services;2021. https://www.nhlbi.nih.gov/health-topics/study-quality-assessment-tools
2. Critical Appraisal Skills Programme. CASP Qualitative Studies Checklist [online]; 19/05/2021 2019;
3. Hong QN, Pluye P, Fàbregues S, et al. Mixed Methods Appraisal Tool (MMAT). Canadian Intellectual Property Office; 2018;
