# Supplementary material for: Blended Psychological Therapy for the Treatment of Psychological Disorders in Adult Patients: Systematic Review and Meta-Analysis
Source: Interact J Med Res. 2024 Oct 29;13:e49660. doi: 10.2196/49660 (PMC11558224; doi:10.2196/49660)
Supplement: Multimedia Appendix 4 [file ijmr_v13i1e49660_app4.pdf]

F2F: face-to-face OL: onlineBT: Blended Therapy CBT: Cognitive-Behavioural Therapy BA: Behavioural Activation iCBT: online CBT HIT: High Intensity Therapy

**BT structure & content described**

| Authors | BT CONCEPT | F2F STRUCTURE | OL STRUCTURE | F2F CONTENT | OL CONTENT |
|---|---|---|---|---|---|
| Askjer et al 2021[1] | Elements of iCBT and F2F CBT incorporated in a session plan where OL modules supplement traditional sessions, often in an alternating sequence. | Starts the treatment<br><br>Usual CBT structure (i.e. psychoeducation, assessment, and problem clarification; behavioral activation; cognitive restructuring; problem solving; and relapse prevention) | Usual CBT structure<br><br>Computer at the clinic if patients not able to do it from home<br><br>Modules in predetermined order; 6 mandatory modules 2 optional - patient & therapist decide which optional modules to add | Core CBT components | Video, audio, interactive exercises, calendar, pdf summaries<br><br>Introductory module<br>Core CBT components make the mandatory modules<br><br>Optional modules: coping with rumination and restructuring of core beliefs<br><br>Reminders (activities; homework) |
| Berger et al, 2018[2] | OL program as an adjunctive tool to use between F2F sessions; no explicit integration of the OL intervention in the treatment - clinical judgment to decide how best to combine/ integrate individual psychotherapy with the OL program. | No specific guidelines/protocol<br><br>Therapists & patients discuss OL content during F2F | Completion at patients' own pace, repeated access to modules allowed<br><br>Auto-Reminders to OL content<br><br>Therapists track patients progress & activity online | nr - Usual CBT | 10 main modules +1 summary module<br>Usual CBT content plus elements from other psychological therapy approaches<br><br>Modules simulate dialogues on concepts & techniques and ask users to select from response options, which will tailor subsequent content.<br><br>Mood assessments; exercises; texts; drawings; pictures; animations and audio clips; printable summaries; worksheets |

F2F: face-to-face OL: onlineBT: Blended Therapy CBT: Cognitive-Behavioural Therapy BA: Behavioural Activation iCBT: online CBT HIT: High Intensity Therapy

| Authors | BT CONCEPT | F2F STRUCTURE | OL STRUCTURE | F2F CONTENT | OL CONTENT |
|---|---|---|---|---|---|
| Bisson et al, 2022[3] | Combines the use of self-help materials (e.g., workbook; website) & regular guidance from mental health professional. 1st session FTF; fortnightly F2F 30-min meetings, may be deliverable via internet/phone, per participant preference. The programme aims to reduce contact time with the therapist. | F2F supplementary<br>Manualised<br>Starts treatment.<br>1st session: 1 hour to develop rapport<br><br>Participant also receives four brief telephone calls or email contacts between sessions to discuss progress, identify any problems that have arisen, and agree new goals. | Online main component<br>Manualised; self-paced<br>Accessible through PC, laptop, tablet, smartphone (via app).<br><br>The programme is interactive and user input dictates feedback to key activities within the programme. Therapist-participant email contacts between sessions<br><br>Participant online activities visible at clinician dashboard. Therapist Log-in to clinician dashboard.<br><br>Eight steps, self-help programme. Later steps relying on mastery of techniques taught in earlier steps. Each step activates a tool in the Toolkit area.<br><br>Branching screens allow user some control to navigate each step, and bookmarking enables program re-entry.<br><br>Online modules accompanied by homework.<br>Sessions are supplemented with assignments to complete between F2F sessions. | 1st session: discuss participant's trauma; log in details; and describe/ demonstrate programme | emails discuss progress, identify any problems that have arisen, and agree new goals.<br><br>Therapist reviews activities on dashboard for discussion during the guidance session.<br><br>Cognitive therapy for PTSD (CT-PTSD) used. The eight steps cover psychoeducation, grounding techniques, management of anxiety, behavioural activation, imaginal exposure, cognitive restructuring, in vivo exposure, and prevention of relapse. Each step provides psycho-education and the rationale for specific components of treatment.<br><br>Content and activities online are audio narrated with keywords and images displayed on screen.<br><br>The programme includes four characters with PTSD, and video content follows their progress through each step of the programme.<br><br>Toolkit area allows access to programme components and information input by users. The tools help participants engage with core CBT-TF techniques |

F2F: face-to-face OL: onlineBT: Blended Therapy CBT: Cognitive-Behavioural Therapy BA: Behavioural Activation iCBT: online CBT HIT: High Intensity Therapy

| **Authors** | **BT CONCEPT** | **F2F STRUCTURE** | **OL STRUCTURE** | **F2F CONTENT** | **OL CONTENT** |
|---|---|---|---|---|---|
| Cloitre et al, 2022[4] | Integrates F2F therapy with internet approaches. Continued therapist input along internet self-help for flexibility and personalization. Web-based program concurrent with F2F session via video conferencing. | F2F supplementary<br><br>Manual with instructions for each session, describing sessions' topics and content<br><br>Crisis or motivational sessions can be F2F (up to 20 min) | Order of components unclear; assumed online session starts treatment. Online module main component<br><br>Access via log on to online portal | Overall goal of the sessions: to reinforce self-directed work on online material; to clarify key concepts presented in the modules; to reinforce engagement with the material; to help the participant tailor the skills; and to support completing modules on a weekly basis. | Trauma-informed program with 10 modules<br><br>Text, video, and audio delivery of psychoeducation, as well as interactive exercises and worksheets to aid the patient in learning and practicing the material |
| Duffy et al, 2020[5] | A therapeutic package for HIT. Patients offered iCBT before commencing F2F. If appointment for f2f (HIT) became available, F2F offered to begin with. | nr | Starts therapy; self-access log-in<br><br>Suggestions for module completion – modules accessible at any time; new modules unlocked as user completes module<br><br>Therapists checks progress & sets weekly feedback time<br><br>Supplementary apps; webpage interface editing; users can respond to content and comment & share tips/ ideas moderated by therapist | nr<br>"counselling" | Usual CBT<br><br>Welcome to the program message<br><br>Interactive elements and graphical exercises; range of satellite applications; journal application & therapeutic writing exercises<br><br>E-mails and/or content shared within the platform (e.g. completed exercises and comments) can be shared with supporter. Anonymous comments & likes from others visible in the system.<br><br>Clinician feedback & responding to the work completed. |

F2F: face-to-face OL: onlineBT: Blended Therapy CBT: Cognitive-Behavioural Therapy BA: Behavioural Activation iCBT: online CBT HIT: High Intensity Therapy

| Authors | BT CONCEPT | F2F STRUCTURE | OL STRUCTURE | F2F CONTENT | OL CONTENT |
|---|---|---|---|---|---|
| Etzelmueller et al, 2018[6] | Video-based synchronous therapy (VTS) sessions + online self-help treatment modules with integrated read-aloud function + online & smartphone based monitoring of behaviour and symptoms | Diagnostic interview starts process<br><br>Patient participated from home<br><br>VTS structured equal to CBT outpatient psychotherapy in Germany<br><br>Results from weekly PHQ-9 discussed within the VTS | OL clustered in blocks - individual lessons on specific, tailored therapeutic content. Therapist decide on content to use and could use online materials to work on during a session (often using a shared screen) or as homework.<br><br>Participants invited to reflect on experiences at the beginning of each lesson<br><br>System messaging function | nr<br>Usual CBT | Usual CBT content<br>Essential + additional modules<br><br>Activity modules; diaries – week planning; mood; activity; emotions; self-care<br><br>Lessons consist of text, exercises and testimonials; also include interactive elements such as audio and video clips<br><br>Homework assignments integrate acquired strategies and techniques into daily life<br><br>Content tailored to responses towards multiple choice options re content<br><br>Messaging between patient & therapist |
| Høifødt et al, 2013[7] | Guided self-help intervention with Web-based CBT program; brief F2F support (15-30 min) after each OL module; and tailored emails between sessions | 1st session: intro to program & instructions<br><br>F2F support after each OL session; flexible intervals between sessions<br><br>Script with 3 compulsory topics per consultation | OL main module<br><br>Self-access log-in<br><br>Weekly modules (5) + workbook<br><br>Motivating emails between sessions | Monitoring and discussion of depression symptoms<br><br>Focus on topics and exercises on each module & participants' practice<br><br>Introduction to next module and motivating patients to adhere to treatment | 5 cognitive behavioural training modules<br><br>Personal workbook (exercises & assessments integrated into each module) that records/updates user's responses; interactive game; feedback evaluation form; emails introducing the next module – some contained brief advice on how to overcome depressive symptoms |
| Jacmon et al, 2009[8] | A combination of internet-based CBT & as-needed in-person individual CBT | F2F sessions scheduled as needed or after all OL sessions done | Protocolised session sequence OL module starts treatment. Unlimited modules completed at own pace<br><br>Self-access log-in; therapist tracking; weekly emails | F2F to address "sticking points" or if depression deteriorated to the extent that patients' ability to apply the skills learned became impaired | Usual CBT content – reading text/exercises; mood monitoring; relaxation training<br><br>Leisure sites including games, humour and music - to reward the participant's efforts with enjoyable activities |

F2F: face-to-face OL: onlineBT: Blended Therapy CBT: Cognitive-Behavioural Therapy BA: Behavioural Activation iCBT: online CBT HIT: High Intensity Therapy

| **Authors** | **BT CONCEPT** | **F2F STRUCTURE** | **OL STRUCTURE** | **F2F CONTENT** | **OL CONTENT** |
|---|---|---|---|---|---|
| Kemmeren et al, 2019[9] | FTF/OL sessions alternated, delivered through internet-based treatment platform. | F2F always starts therapy<br><br>1st session: Instructions for BT<br><br>F2F followed OL content; content/ exercises discussed during F2F | Self-access log-in<br><br>After 1st F2F, 2 mandatory modules (therapist-guided); remaining modules unlocked one at a time – therapists to agree before activating module. Modules done in between F2F sessions<br><br>Daily mood tracking – therapists patients can track progress & activities. Therapist feedback in between F2F. | F2F to be used for reflection on mood ratings; previous module review; repeating/ clarifying online exercises and lessons; deepening personal themes; deciding/ discussing next OL module | Usual CBT<br>Modules start with a description of the goal and content of the therapeutic approach and an illustrative video<br><br>Mood graph; calendar; text modules & exercises; reading material with illustrations, examples, and tips; interactive homework exercise; messaging system; therapist written feedback<br><br>All modules end with a summary followed by an evaluation questionnaire, and a depressive symptoms assessment |
| Kenter et al. 2013[10] | If used, OL before F2F treatment. After 5 weeks, regular F2F treatment received. | nr<br><br>Starts after 5 weeks of OL | Therapy starts with OL<br><br>Support/feedback by email within 3 working days | nr | Content includes instructions, exercises, examples for applying PST principles<br>The program consists of solving problems in six steps – the last step is writing a plan.<br>Feedback consists of guidance through treatment plus personalised feedback |
| Kenter et al. 2015[11] | OL treatment sessions could be offered prior, simultaneously, or after the FTF treatment, depending on the needs of the patient/ preferences of therapist. | During F2F sessions CBT was offered and OL activities discussed. | 4-5 core modules, CBT-based<br><br>Patients complete 1-2 exercises of a module per week in no particular order<br><br>OL exercises with therapist support; personalized written feedback. | Nr<br><br>Online activities discussed during F2F sessions | Disorder specific core CBT modules – could be supplemented with exercises<br><br>Thought records; mood diary and/or activity diary; anxiety hierarchy and relaxation exercises<br><br>Personalized written feedback and motivational techniques |

F2F: face-to-face OL: onlineBT: Blended Therapy CBT: Cognitive-Behavioural Therapy BA: Behavioural Activation iCBT: online CBT HIT: High Intensity Therapy

| Authors | BT CONCEPT | F2F STRUCTURE | OL STRUCTURE | F2F CONTENT | OL CONTENT |
|---|---|---|---|---|---|
| Kok et al., 2014[12] | Internet-based guided self-help program based on exposure therapy with weekly support followed by face-to-face psychotherapy | nr | Therapy starts with OL<br><br>Adapted from book, elements of psychoeducation & exposure therapy – exposure exercises as homework<br><br>Modules locked until patient reports on achievements & receives feedback. Materials accessible & can be revisited throughout waiting list period<br><br>Standardised reminders & auto-emails re online activities | nr | Background information; psychoeducation on phobias; information on relapse prevention; recommendations and support sources. Patients' examples illustrate principles/concepts of the intervention.<br><br>Patients select fear-inducing situations or stimuli and construct a ‘‘fear hierarchy’ and planning monitored by the coach<br><br>Supportive message from coach 1x/week |
| Kooistra et al 2016[13] | F2F/OL integrated into one therapy protocol. Fixed alternate sequence of F2F/OL components. Patients work though part of the protocol autonomously & at home. | F2F starts treatment<br><br>15 min discussion at each session start<br><br>Fixed sequence, usual CBT structure<br><br>Protocolised session sequence | Usual CBT structure; fixed, protocolised session sequence<br><br>OL instructions on platform use<br>Mandatory & optional exercises<br><br>Therapist feedback after each session<br>Email reminders to encourage patients to access OL platform and engage in treatment. | 1st session: explanations re BT + OL environment<br><br>Usual CBT - focus on adapting therapy content to patient's needs, e.g. by practising skills in role plays; identifying core problems<br><br>Discussing issues from previous week & review homework / OL session activity | Information on how to work with the platform<br>OL sessions elaborate on F2F themes<br>Psycho-education (written information + short video in lay terms)<br>Homework illustrated by vignettes of fictional patients<br>Background information & mood diary<br>Open-ended question for commenting on the online sessions and to promote reflective thinking on homework<br>Email reminders re mood ratings & homework; therapist feedback |

F2F: face-to-face OL: onlineBT: Blended Therapy CBT: Cognitive-Behavioural Therapy BA: Behavioural Activation iCBT: online CBT HIT: High Intensity Therapy

| **Authors** | **BT CONCEPT** | **F2F STRUCTURE** | **OL STRUCTURE** | **F2F CONTENT** | **OL CONTENT** |
|---|---|---|---|---|---|
| Kooistra et al 2019[14] | Protocol-based, semi-structured, evenly distributed (1:1) F2F sessions & web-based (online) weekly sessions (fixed order) | Fixed, protocolised session sequence, usual CBT structure – personalisation allowed for session themes/ techniques<br><br>Treatment starts & ends with F2F; OL instructions given during 1st F2F | Protocolised, fixed weekly session sequence, usual CBT structure<br><br>OL sessions done at home, self-access log in<br><br>Therapist feedback after each OL via platform<br><br>Once completed, OL session could be repeated if warranted by therapist; OL still accessible after treatment | Usual CBT<br><br>Therapists assessed & monitored depression severity changes in session | Usual CBT<br><br>Short video in each OL session in which a therapist explains the theory in lay terms; testimonials from 2 fictional patients instruct on exercises.<br><br>Information reinforces and develops on content of the face-to-face sessions. Homework exercises (e.g. monitoring activities, feelings, thoughts, behaviour)<br><br>OL therapeutic feedback provided on content & progress |
| Kooistra et al 2020[15] | Protocol-based, semi-structured, evenly distributed (1:1) F2F sessions & web-based (online) weekly sessions (fixed order) | F2F starts treatment. Sessions in a fixed order, based on CBT manuals<br><br>Therapists advised to plan weekly sessions but allowed to deviate from manual when necessary<br><br>Patients planned sessions with therapist, focussing on a domain of the CBT protocol | Sessions in a fixed order; therapists allowed to repeat an online session<br><br>Patients and therapists logged into a secure website<br><br>Asynchronous written therapeutic feedback message per completed online session – patients able to access to the next online session after reading feedback | Usual CBT content | OL content corresponded with previous face-to-face session's content<br><br>Text, short videos, images, patient vignettes, and homework exercises<br><br>Daily mood diary, weekly monitoring of depression severity (QIDS-SR), and a messaging function to contact therapists<br><br>Written therapist feedback |

F2F: face-to-face OL: onlineBT: Blended Therapy CBT: Cognitive-Behavioural Therapy BA: Behavioural Activation iCBT: online CBT HIT: High Intensity Therapy

| Authors | BT CONCEPT | F2F STRUCTURE | OL STRUCTURE | F2F CONTENT | OL CONTENT |
|---|---|---|---|---|---|
| Lungu et al, 2020[16] | Video-based sessions with therapist. Regular therapist-led CBT sessions with iCBT modules into an integrated treatment. Online tools for clients to use in between sessions – therapists could personalise digital tools in several ways. | Video F2F conducted on video platform with separate portals & customized functions for clients and therapists – video recording possible upon client consent<br><br>Platform enabled therapists to conduct tasks before, during, after, and in between therapy sessions in a single environment | Activities selected and personalised from a pool of standardised materials. OL materials assigned to be used between sessions / revisited<br><br>Lessons and exercises to be completed before each F2F session in sequence - freely accessible afterwards<br><br>Therapist & patient tracking and asynchronous messaging system; alerts/reminders re lessons/ exercises; therapy notes and feedback | During sessions, therapists could share their screen to collaboratively complete digital exercises with clients or preview specific exercises and digital lessons they intended to assign to clients<br><br>"virtual rehearsal" of a desired new behaviour possible (such as completing a psychotherapy digital exercise) | Transdiagnostic treatment approaches, such as the Unified Treatment Protocol, ACT, DBT<br><br>Digital lessons with storytelling approach through animated videos and quizzes<br><br>Principles and skills taught in the digital lessons and exercises included content from different therapy approaches<br><br>Therapists tracking - review clients' completion of assigned practices, clinical outcomes; and other communications; therapist feedback. |
| Ly et al, 2015[17] | Smartphone activity scheduling application in addition to face-to-face treatment | Protocolised session sequence | Instructions for Smartphone app use sent by email before 1st F2F session<br><br>Back-end system for therapist tracking re patients' progress & activity; to send messages<br><br>Weekly homework online | Behaviour discussed during F2F; identification of individualized treatment targets, monitoring and scheduling activities<br><br>Usual BA (Psychoeducation; treatment rationale; activity plan; homework; follow-up on homework/activity plan; set relapse prevention plan) | Behaviours database – patients could pick & add behaviours to their app; could provide suggestions and add their own behavioural activities between sessions<br><br>Statistics and summaries of quantitative (e.g., behaviour frequency) and qualitative data (e.g., comments)<br><br>Personalised messages from therapist; weekly general educational messages |

F2F: face-to-face OL: onlineBT: Blended Therapy CBT: Cognitive-Behavioural Therapy BA: Behavioural Activation iCBT: online CBT HIT: High Intensity Therapy

| Authors | BT CONCEPT | F2F STRUCTURE | OL STRUCTURE | F2F CONTENT | OL CONTENT |
|---|---|---|---|---|---|
| Månsson et al, 2013[18] | Blended iCBT with F2F CBT. No computerized treatment components; the main part of the system consisted of digital material that in a F2F session would have been presented on printed paper or verbally. | nr | Access at home & in the clinic. Self-access log-in<br><br>Therapist and patient decided on platform use during therapy and/or in contact between sessions. Therapists controlled access to support resources and information<br><br>Communications via platform – memos on topics; uploading & sharing personal files | Usual CBT content | Materials corresponded to that in a face-to-face session presented on printed paper or verbally<br><br>Platform with basic components of CBT, such as scheduling visits; agenda; setting goals; questionnaires and forms used in homework assignments<br><br>Platform library with text and media resources for use in psychoeducation and as homework assignments and/or part of the F2F treatment; memos; personal files |
| Månsson et al, 2017[19] | Internet-based support system used as an adjunct to conventional CBT delivered F2F. Designed to support the delivery of F2F CBT and not to replace in-session treatment activities. | NO protocolised manual | Self-access log-in<br>Communication between therapy sessions; media sharing media; clarifying homework assignments<br><br>Content, tailored to patients' needs – materials to be used as part of F2F treatment as 'online handouts'.<br>Writing /storing memos | nr<br>CBT sessions tailored according to patient's needs | Homework assignments; activity progress; assessment forms; treatment goals; agenda; own therapy-related information (text; images) in personal library; audio files; notes, messages & memos<br><br>Library with text documents; audio (e.g. relaxation instructions) and movies.<br>Online handouts contained supplemental information on CBT (e.g. daily thought records; sleep diaries) |
| Mol et al, 2018[20] | Integrated, standardized CBT treatment protocol combining F2F sessions and digital modules (1:2 ratio) | nr | OL protocolised sessions – therapists could deviate from the protocol by repeating online sessions<br><br>Feedback message after each completed online session; patient/therapist messaging | not described, usual CBT | Usual CBT core components<br><br>Diary and questionnaires to monitor symptoms. Feedback msg from therapist.<br><br>Messages re practical issues (eg, upcoming appointments; reminders or questions re assignments) |

F2F: face-to-face OL: onlineBT: Blended Therapy CBT: Cognitive-Behavioural Therapy BA: Behavioural Activation iCBT: online CBT HIT: High Intensity Therapy

| Authors | BT CONCEPT | F2F STRUCTURE | OL STRUCTURE | F2F CONTENT | OL CONTENT |
|---|---|---|---|---|---|
| Nakao et al, 2018[21] | CBT sessions delivered by therapist and computer sessions integrated into one treatment protocol. | Protocolised session sequence – during the 1st session a guidebook with instructions was discussed. Sessions were tailored to patient – included reviewing OL material; discussing problem; practicing CBT skills; setting homework | Self-access to OL<br><br>Patients work at own pace<br><br>Protocolised session sequence | Usual CBT content<br><br>Guidebook offers information on how and when to use the specific Web-based content to meet individual needs of patients | Usual CBT<br><br>Psychoeducational video clips and short texts<br><br>Rating/monitoring daily mood graphs<br><br>Entering text as guided on the Web screen to assist mastering CBT skills |
| Romijn G, et al., 2021[22] | Combines F2F treatment with Internet components into one integrated treatment protocol. Part of the F2F treatment is replaced by OL component, while traditional F2F relationship between therapist and patient is retained. | Protocolised session sequence – 1st session is F2F<br><br>Session 1: psychoeducation Following sessions: discussing previous / prepping next OL session | Protocolised session sequence; self log-in<br><br>OL sessions to accessed once, could be repeated<br><br>OL therapist feedback on assignments/homework<br><br>Scheduling next F2F sessions | Usual CBT; psychoeducation used for explanation of disorder; treatment; blended approach<br><br>Separate manualised protocols used for panic disorder, social anxiety disorder and GAD, with specific content for anxiety disorders (e.g. CBT and exposure therapy) | Usual CBT - sessions' content sequence introduces/complements/practices F2F content<br><br>Information (videos and text), testimonials from fictional patients, assignments and homework exercises<br><br>Default text templates for feedback and instructions for every online session as a therapist aid for feedback – therapists could tailor texts to clients'' specific needs |
| Tarp et al, 2022[23] | Combines face-to-face and internet-based therapy into one integrated protocol. The content of the blended protocol is similar to the face-to-face AUD treatment offer, based on evidence-based manuals for MI and CBT | No firm structure describing a fixed number of F2F sessions before offering patients to continue with OL modules – therapists encouraged to discuss with patients and decide on the optimal blend. | The internet-based platform consists of 4 modules, which the therapist can gradually add to patients' individual platform. Each module has submodules with a fixed structure. Therapist decides on homework assignments with patient<br><br>Patients' progress tracker & therapists feedback features. Patients can use the platform | Standard face-to-face treatment in Danish alcohol treatment clinics, based on evidence-based manuals for MI and CBT | Content similar to F2F AUD treatment offer, based on evidence-based manuals for MI and CBT; elements are similar to the CBT modules in the manual used in other project (12-step facilitation, MI).<br><br>Text boxes and videos with information and assignments. Patients receive online feedback from their therapist on assignments. |

F2F: face-to-face OL: onlineBT: Blended Therapy CBT: Cognitive-Behavioural Therapy BA: Behavioural Activation iCBT: online CBT HIT: High Intensity Therapy

| Authors | BT CONCEPT | F2F STRUCTURE | OL STRUCTURE | F2F CONTENT | OL CONTENT |
|---|---|---|---|---|---|
| | | Standard F2F using CBT +MI | anytime, even after completing treatment | | Therapy information; multiple exercises and homework assignments; training in optional skills according to patients' individual needs; relapse prevention. |
| Thase et al. 2018[24] | Integrates computer-delivered training with therapist support, reducing the amount of therapist time and effort | 1st session F2F - explanation of CBT treatment and OL component<br><br>Abbreviated time of sessions – 25 min F2F + booster sessions | Usual CBT structure in a 9-module multimedia program – modules' use unlimited, accessible at any time and work at own pace<br><br>Therapist can track patients progress & activity | Therapists review materials covered in the module and self-help assignments as a springboard for applying CBT methods to specific problem areas identified by the patient | Usual CBT - specific content from the program provided at each session Psychoeducation from a psychiatrist-narrator; feedback to users<br><br>Video illustrations; mood graphs; interactive skill-building exercises; quizzes |
| van de Wal et al 2017[25] | Website provides CBT online materials and the option to engage in therapist-patient interaction. Blended sessions unevenly distributed with both components integrated. | Protocolised session sequence - 1st session 90 min, comprising case conceptualisation & formulation<br><br>Usual CBT structure, FCR-focussed | Self-access to website, accessible throughout the entire therapy as it runs parallel to f2f sessions<br><br>e-consultations supplementary to F2F – could be substituted by workbook + 15 min phone consultation | Usual CBT, including mindfulness or relaxation exercises<br><br>Therapy process evaluation<br><br>Booster session to review FCR model and progress made during therapy; relapse prevention plan | Website contains over 70 pages of content, including information (10 scripts), at-home assignments (27 tasks), assessments (6 tests), audio (2 clips) and video (15 fragments). An incorporated library includes additional information on cancer-related topics<br><br>Paper workbook/DVD/CD also available |
| Vernmark et al, 2019[26] | F2F and OL integrated and used in conjunction with each other within the same time period | nr | Protocolised, self-accessible, weekly (fixed order) session sequence - modules supplied by therapist<br>Weekly OL therapist feedback between F2F sessions | Usual CBT | Usual CBT<br><br>exercises - texts; images; progress monitoring & communications |

F2F: face-to-face OL: onlineBT: Blended Therapy CBT: Cognitive-Behavioural Therapy BA: Behavioural Activation iCBT: online CBT HIT: High Intensity Therapy

| Authors | BT CONCEPT | F2F STRUCTURE | OL STRUCTURE | F2F CONTENT | OL CONTENT |
|---|---|---|---|---|---|
| Witlox et al, 2021[27] | OL/F2F components are integrated in the treatment | Protocolised session sequence – 1st session: discussing complaints; instructions to OL sessions | Protocolised session sequence Self-access log-in<br><br>Modules based on ACT-module adapted from book<br><br>Progress monitoring | Psycho-education;<br>if required, short CBT or PST | Six core processes of ACT<br><br>text, video & audio files to deliver information and exercises<br><br>Daily mindfulness practice & motivational messages |
| Wu, Chen, Wickham, O'Neil-Hart, Chen & Lungu, 2021[28] | F2F sessions with coaches (in person or via teletherapy) with digital activities to introduce/reinforce key coaching concepts and skills. | Coaches used secure, web-based platform to see clients via video conference, access assessment data, and assign and review digital activities. | Flexible, platform-based OL component –pre-set, time-fixed *or* personalised content and activities assigned based on F2F discussions<br><br>Coach feedback via asynchronous messaging as needed | Core principles of therapy approaches used (eg, acceptance, values), self-practice/ self-reflection of these principles and skills | Video lessons on core CBT concepts and skills through a storytelling approach. Digital exercises akin to digitized versions of standard CBT worksheets. Activities could be personalized and assigned for use between sessions - video lessons and exercises derived from CBT, DBT, ACT<br><br>Coach feedback on completed exercises |
| Wu,Wickham, Chen, Chen & Lungu, 2021[29] | Therapy sessions via video-based teletherapy with digital components of care that cover key therapeutic concepts and skills. | Providers conducted therapy sessions via video-based Teletherapy<br><br>During F2F, personalised, digital activities are assigned | Digital activities through the platform based on presenting issues. Completion of digital activities monitored in real time via platform by providers<br><br>Therapist-led reminders for completion of assignments<br><br>Online activities completion asynchronous & at own pace | During F2F sessions therapists promote personalized care; discussions re therapy content in depth, and check on homework completion<br><br>Short-term, goal-based model employed based on principles from CBT, DBT and ACT | Digital video lessons with a storytelling approach, presenting key CBT-based concepts and skills. Brief quiz at the end of each video lesson.<br><br>Digital exercises analogous to paper logs or handouts for practicing skills<br><br>Exercise responses can be viewed in real time by both the provider and the client, and the provider is able to comment on it |

F2F: face-to-face  OL: onlineBT: Blended Therapy  CBT: Cognitive-Behavioural Therapy  BA: Behavioural Activation  iCBT: online CBT  HIT: High Intensity Therapy

**Online content per study**

| Authors | Protocolised /manualised | CBT Framework | Usual CBT content | | | | Synchro-nous online content | Audio | Video | Written text | Monitoring (Exercises/ Diaries) | Assessments & Questionnaires | Messaging | Images | Gaming | Digital therapist feedback |
|---|---|---|---|---|---|---|---|---|---|---|---|---|---|---|---|---|
| | | | BA/ training | Psycho-education | Relapse Prevention | Cognitive restructuring | | | | | | | | | | |
| Askjer et al 2021[1] | - | ✓ | ✓ | ✓ | ✓ | ✓ | - | ✓ | ✓ | ✓ | ✓ | ✓ | - | - | - | - |
| Berger et al, 2018[2] | N | ✓ | ✓ | ✓ | - | ✓ | - | ✓ | - | ✓ | - | ✓ | - | ✓ | - | - |
| Bisson et al, 2022[3] | ✓ | ✓ | ✓ | ✓ | ✓ | ✓ | - | ✓ | ✓ | ✓ | ✓ | - | - | ✓ | - | ✓ |
| Cloitre et al, 2022[4] | ✓ | ✓ | - | ✓ | - | - | ✓ | ✓ | ✓ | ✓ | ✓ | - | - | - | - | - |
| Duffy et al, 2020[5] | - | ✓ | ✓ | - | - | ✓ | - | - | - | - | ✓ | - | - | - | - | ✓ |
| Etzelmueller , 2018[6] | - | ✓ | ✓ | ✓ | ✓ | ✓ | ✓ | ✓ | ✓ | ✓ | ✓ | - | ✓ | - | - | - |
| Høifødt et al, 2013[7] | - | ✓ | ✓ | - | - | ✓ | - | - | - | - | ✓ | ✓ | - | - | ✓ | - |
| Jacmon et al, 2009[8] | ✓ | ✓ | ✓ | ✓ | - | ✓ | - | - | - | - | ✓ | - | - | - | ✓ | - |
| Kemmeren et al, 2019[9] | - | ✓ | ✓ | ✓ | ✓ | ✓ | - | - | ✓ | ✓ | ✓ | ✓ | ✓ | ✓ | - | ✓ |
| Kenter et al. 2013[10] | - | PST | - | ✓ | - | - | - | - | - | - | ✓ | - | - | - | - | ✓ |
| Kenter et al. 2015[11] | - | ✓ | ✓ | ✓ | ✓ | ✓ | ✓ | - | - | - | ✓ | - | - | - | - | ✓ |
| Kok et al., 2014[12] | - | Psycho-therapy | ✓ | ✓ | ✓ | - | - | - | - | - | ✓ | - | ✓ | - | - | ✓ |
| Kooistra et al 2016[13] | ✓ | ✓ | ✓ | ✓ | ✓ | ✓ | - | - | ✓ | - | ✓ | - | - | - | - | ✓ |
| Kooistra et al 2019[14] | ✓ | ✓ | ✓ | ✓ | ✓ | ✓ | - | - | ✓ | - | ✓ | - | ✓ | - | - | ✓ |
| Kooistra et al 2020[15] | ✓ | ✓ | ✓ | ✓ | ✓ | ✓ | - | - | ✓ | ✓ | ✓ | - | ✓ | ✓ | - | ✓ |
| Lungu et al, 2020[16] | - | ✓+ other (UTP, ACT, DBT) | ✓ | ✓ | - | ✓ | ✓ | - | ✓ | - | ✓ | ✓ | ✓ | - | - | ✓ |
| Ly et al, 2015[17] | ✓ | BA | ✓ | ✓ | ✓ | - | - | - | ✓ | ✓ | ✓ | - | ✓ | - | - | ✓ |

F2F: face-to-face OL: onlineBT: Blended Therapy CBT: Cognitive-Behavioural Therapy BA: Behavioural Activation iCBT: online CBT HIT: High Intensity Therapy

| Authors | Protocolised /manualised | CBT Framework | Usual CBT content | | | | Synchro-nous online content | Audio | Video | Written text | Monitoring (Exercises/ Diaries) | Assessments & Questionnaires | Messaging | Images | Gaming | Digital therapist feedback |
|---|---|---|---|---|---|---|---|---|---|---|---|---|---|---|---|---|
| | | | BA/ training | Psycho-education | Relapse Prevention | Cognitive restructuring | | | | | | | | | | |
| Månsson et al, 2013[18] | - | ✓ | ✓ | ✓ | - | ✓ | - | ✓ | - | ✓ | - | ✓ | ✓ | - | - | ✓ |
| Månsson et al, 2017[19] | N | ✓ | ✓ | ✓ | - | ✓ | - | ✓ | - | ✓ | - | ✓ | ✓ | ✓ | - | ✓ |
| Mol et al, 2018[20] | ✓ | ✓ | ✓ | ✓ | ✓ | ✓ | - | - | - | - | ✓ | ✓ | ✓ | - | - | ✓ |
| Nakao et al, 2018[21] | ✓ | ✓ | ✓ | ✓ | ✓ | ✓ | - | - | ✓ | ✓ | ✓ | ✓ | - | - | - | - |
| Romijn G, et al., 2021[22] | ✓ | ✓ | ✓ | ✓ | ✓ | ✓ | - | - | ✓ | ✓ | ✓ | - | - | - | - | ✓ |
| Tarp et al, 2022[23] | ✓ | ✓+ other (MI) | ✓ | ✓ | ✓ | ✓ | - | - | ✓ | ✓ | ✓ | - | - | - | - | ✓ |
| Thase et al., 2018[24] | - | ✓ | ✓ | ✓ | ✓ | ✓ | - | ✓ | ✓ | - | ✓ | ✓ | - | ✓ | - | ✓ |
| van de Wal et al 2017[25] | ✓ | ✓ | ✓ | ✓ | ✓ | ✓ | ✓ | ✓ | ✓ | - | ✓ | ✓ | - | - | - | - |
| Vernmark et al, 2019[26] | ✓ | ✓ | ✓ | ✓ | ✓ | ✓ | - | - | - | ✓ | ✓ | - | - | ✓ | - | ✓ |
| Witlox et al, 2021[27] | ✓ | ACT | - | ✓ | - | - | - | ✓ | ✓ | ✓ | ✓ | - | ✓ | - | - | - |
| Wu, Chen et al, 2021[28] | - | ✓+ other (DBT, ACT) | - | ✓ | - | - | - | - | ✓ | - | ✓ | ✓ | - | - | - | - |
| Wu, Wickham et al, 2021[29] | - | ✓ + other (DBT, ACT) | - | ✓ | - | - | - | - | ✓ | - | ✓ | ✓ | - | - | - | - |
| TOTAL # studies | 14 | 25 | 24 | 27 | 17 | 22 | 5 | 10 | 18 | 15 | 25 | 13 | 11 | 7 | 2 | 18 |

**Key**

ACT = Acceptance & Commitment Therapy
BA = Behavioural Activation
CBT = Cognitive-Behavioural Therapy
DBT = Dialectical-Behavioural Therapy
MI = Motivational Interview
N = intervention explicitly described as non-protocolised
PST = Problem-Solving Therapy
UTP = Unified Transdiagnostic Protocol

F2F: face-to-face OL: onlineBT: Blended Therapy CBT: Cognitive-Behavioural Therapy BA: Behavioural Activation iCBT: online CBT HIT: High Intensity Therapy

## References


1. Askjer S, Mathiasen K. The working alliance in blended versus face-to-face cognitive therapy for depression: A secondary analysis of a randomized controlled trial. Internet Interv; Sep 2021;25:100404. doi:10.1016/j.invent.2021.100404
2. Berger T, Krieger T, Sude K, Meyer B, Maercker A. Evaluating an e-mental health program ("deprexis") as adjunctive treatment tool in psychotherapy for depression: Results of a pragmatic randomized controlled trial. J Affect Disord; Feb 2018;227:455-462. doi:10.1016/j.jad.2017.11.021
3. Bisson JI, Ariti C, Cullen K, et al. Guided, internet based, cognitive behavioural therapy for post-traumatic stress disorder: pragmatic, multicentre, randomised controlled non-inferiority trial (RAPID). BMJ; Jun 16 2022;377:e069405. doi:10.1136/bmj-2021-069405
4. Cloitre M, Amspoker AB, Fletcher TL, et al. Comparing the Ratio of Therapist Support to Internet Sessions in a Blended Therapy Delivered to Trauma-Exposed Veterans: Quasi-experimental Comparison Study. JMIR Ment Health; Apr 27 2022;9(4):e33080. doi:10.2196/33080
5. Duffy D, Enrique A, Connell S, Connolly C, Richards D. Internet-Delivered Cognitive Behavior Therapy as a Prequel to Face-To-Face Therapy for Depression and Anxiety: A Naturalistic Observation. Front Psychiatry; 09 Jan 2020;10:902. 902. doi:10.3389/fpsyt.2019.00902
6. Etzelmueller A, Radkovsky A, Hannig W, Berking M, Ebert DD. Patient's experience with blended video- and internet based cognitive behavioural therapy service in routine care. Internet Interv; Jun 2018;12:165-175. doi:10.1016/j.invent.2018.01.003
7. Hoifodt RS, Lillevoll KR, Griffiths KM, et al. The clinical effectiveness of web-based cognitive behavioral therapy with face-to-face therapist support for depressed primary care patients: randomized controlled trial. Empirical Study; Interview; Quantitative Study. J Med Internet Res; Aug 5 2013;15(8):e153. doi:10.2196/jmir.2714
8. Jacmon J, Malouff JM, Taylor N. Treatment of major depression: Effectiveness of cognitive-behavioural therapy with an internet course as a central component. Empirical Study; Quantitative Study. E J Appl Psychol; 2009;5(2):1-8.
9. Kemmeren LL, van Schaik A, Smit JH, et al. Unraveling the Black Box: Exploring Usage Patterns of a Blended Treatment for Depression in a Multicenter Study. JMIR Ment Health; Jul 25 2019;6(7):e12707. doi:10.2196/12707
10. Kenter R, Warmerdam L, Brouwer-Dudokdewit C, Cuijpers P, van Straten A. Guided online treatment in routine mental health care: an observational study on uptake, drop-out and effects. BMC Psychiatry; Jan 31 2013;13:43. 43. doi:10.1186/1471-244X-13-43
11. Kenter RMF, van de Ven PM, Cuijpers P, et al. Costs and effects of Internet cognitive behavioral treatment blended with face-to-face treatment: Results from a naturalistic study. Internet Interv; 2015;2(1):77-83. doi:10.1016/j.invent.2015.01.001
12. Kok RN, van Straten A, Beekman AT, Cuijpers P. Short-term effectiveness of web-based guided self-help for phobic outpatients: randomized controlled trial. Empirical Study; Interview; Quantitative Study; Treatment Outcome. J Med Internet Res; Sep 29 2014;16(9):e226. doi:10.2196/jmir.3429
13. Kooistra LC, Ruwaard J, Wiersma JE, et al. Development and initial evaluation of blended cognitive behavioural treatment for major depression in routine specialized mental health care. Internet Interv; May 2016;4:61-71. doi:10.1016/j.invent.2016.01.003
14. Kooistra LC, Wiersma JE, Ruwaard J, et al. Cost and Effectiveness of Blended Versus Standard Cognitive Behavioral Therapy for Outpatients With Depression in Routine Specialized Mental Health Care: Pilot Randomized Controlled Trial. J Med Internet Res; Oct 29 2019;21(10):e14261. doi:10.2196/14261
15. Kooistra L, Ruwaard J, Wiersma J, van Oppen P, Riper H. Working Alliance in Blended Versus Face-to-Face Cognitive Behavioral Treatment for Patients with Depression in Specialized Mental Health Care. J Clin Med; Jan 27 2020;9(2)347. doi:10.3390/jcm9020347
16. Lungu A, Jun JJ, Azarmanesh O, Leykin Y, Chen CE. Blended Care-Cognitive Behavioral Therapy for Depression and Anxiety in Real-World Settings: Pragmatic Retrospective Study. J Med Internet Res; Jul 6 2020;22(7):e18723. e18723. doi:10.2196/18723

F2F: face-to-face OL: onlineBT: Blended Therapy CBT: Cognitive-Behavioural Therapy BA: Behavioural Activation iCBT: online CBT HIT: High Intensity Therapy


17. Ly KH, Topooco N, Cederlund H, et al. Smartphone-Supported versus Full Behavioural Activation for Depression: A Randomised Controlled Trial. PLoS One; 2015;10(5):e0126559. e0126559. doi:10.1371/journal.pone.0126559

18. Mansson KN, Skagius Ruiz E, Gervind E, Dahlin M, Andersson G. Development and initial evaluation of an Internet-based support system for face-to-face cognitive behavior therapy: a proof of concept study. J Med Internet Res; Dec 10 2013;15(12):e280. doi:10.2196/jmir.3031

19. Mansson KN, Klintmalm H, Nordqvist R, Andersson G. Conventional Cognitive Behavioral Therapy Facilitated by an Internet-Based Support System: Feasibility Study at a Psychiatric Outpatient Clinic. JMIR Res Protoc; Aug 24 2017;6(8):e158. doi:10.2196/resprot.6035

20. Mol M, Dozeman E, Provoost S, van Schaik A, Riper H, Smit JH. Behind the Scenes of Online Therapeutic Feedback in Blended Therapy for Depression: Mixed-Methods Observational Study. J Med Internet Res; May 3 2018;20(5):e174. doi:10.2196/jmir.9890

21. Nakao S, Nakagawa A, Oguchi Y, et al. Web-Based Cognitive Behavioral Therapy Blended With Face-to-Face Sessions for Major Depression: Randomized Controlled Trial. J Med Internet Res; Sep 21 2018;20(9):e10743. doi:10.2196/10743

22. Romijn G, Provoost S, Batelaan N, Koning J, van Balkom A, Riper H. Does it blend? Exploring therapist fidelity in blended CBT for anxiety disorders. Internet Interv; Sep 2021;25:100418. 100418. doi:10.1016/j.invent.2021.100418

23. Tarp K, Nielsen AS. Patient Perspectives on Videoconferencing-Based Treatment for Alcohol Use Disorders. Empirical Study; Interview; Qualitative Study. Alcohol Treat Q; Oct 2017;35(4):344-358. doi:10.1080/07347324.2017.1348785

24. Thase ME, Wright JH, Eells TD, et al. Improving the Efficiency of Psychotherapy for Depression: Computer-Assisted Versus Standard CBT. Am J Psychiatry; Mar 1 2018;175(3):242-250. doi:10.1176/appi.ajp.2017.17010089

25. van de Wal M, Thewes B, Gielissen M, Speckens A, Prins J. Efficacy of Blended Cognitive Behavior Therapy for High Fear of Recurrence in Breast, Prostate, and Colorectal Cancer Survivors: The SWORD Study, a Randomized Controlled Trial. J Clin Oncol; Jul 1 2017;35(19):2173-2183. doi:10.1200/JCO.2016.70.5301

26. Vernmark K, Hesser H, Topooco N, et al. Working alliance as a predictor of change in depression during blended cognitive behaviour therapy. Cogn Behav Ther; Jul 2019;48(4):285-299. doi:10.1080/16506073.2018.1533577

27. Witlox M, Garnefski N, Kraaij V, et al. Blended Acceptance and Commitment Therapy Versus Face-to-face Cognitive Behavioral Therapy for Older Adults With Anxiety Symptoms in Primary Care: Pragmatic Single-blind Cluster Randomized Trial. J Med Internet Res; Mar 26 2021;23(3):e24366. e24366. doi:10.2196/24366

28. Wu MS, Chen SY, Wickham RE, O'Neil-Hart S, Chen C, Lungu A. Outcomes of a Blended Care Coaching Program for Clients Presenting With Moderate Levels of Anxiety and Depression: Pragmatic Retrospective Study. JMIR Ment Health; Oct 21 2021;8(10):e32100. doi:10.2196/32100

29. Wu MS, Wickham RE, Chen SY, Chen C, Lungu A. Examining the Impact of Digital Components Across Different Phases of Treatment in a Blended Care Cognitive Behavioral Therapy Intervention for Depression and Anxiety: Pragmatic Retrospective Study. JMIR Form Res; Dec 17 2021;5(12):e33452. doi:10.2196/33452
