# Supplementary material for: Blended Psychological Therapy for the Treatment of Psychological Disorders in Adult Patients: Systematic Review and Meta-Analysis
Source: Interact J Med Res. 2024 Oct 29;13:e49660. doi: 10.2196/49660 (PMC11558224; doi:10.2196/49660)
Supplement: Multimedia Appendix 5 [file ijmr_v13i1e49660_app5.pdf]

Uptake & adherence results

Authors	Overall Uptake - n(%)	BT - Uptake n(%)	Overall Dropouts- n(%)	BT Dropouts- n(%)	Overall adherence n(%)	BT adherence n(%)	BT Full treatment n (%)
Askjer & Mathiasen 2021[1]	72/76 (95%)	36/38 (95%)	N=14/72 (19%)	N=10/36 (28%)	nr	adherence criteria nr	nr
Berger et al, 2018[2]	98/98 (100%) (uptake criteria: used OL once)	51/51 (100%)	29/98 (29.6%) (dropout criteria: did not complete 12-weeks (T1) questionnaires	14/51 (27.5%) (criteria: did not complete 12-weeks (T1) questionnaires	nr	37/51 (72.5%)	37/51 (~73%) (criteria for full treatment: completed 12 wks)
Bisson et al, 2022[3]	182/196 (93%)	87/97 (89.7%)	160/182 (88%)	10/87 (11.5%)	132/182 (72.5%) Criteria for adherence: BT= 3< FTF completed Ctrl= 8< or judged to need <8 sessions	77/87 (88.5%)	nr
Cloitre et al, 2022[4]	See BT	171/202 (85%) [BT group 1: 86/101 (85%) BT group 2: 85/101 (84%)]	31/171 (18%)	31/171 (18%)<br><br>[BT group 1: 15/86 (17.4%)<br><br>BT group 2: 16/85 (19%)]	See BT	139/171 (82%) Criteria: completed 5 modules [BT group 1: 72/86 (84%) [BT group 2: 67/85 (79%)]	(100/202) 49.5%<br><br>[BT group 1: 59/101 (58%) BT group 2: 51/101 (51%)]
Duffy et al, 2020[5]	See BT	n= 123/123 (100%)	See BT	45/123 (37%)	See BT	n=78/123 (63%)	68/123 55%
Etzelmueller et al, 2018[6]	See BT	15/25 (60%)	See BT	3/15 (20%)	See BT	12/15 (80%)	na
Høifødt et al, 2013[7]	104/106 (98%)	50/52 (96%)	22/104 (21%)	19/50 (38%)	nr	31/50 (62%) criteria: completed OL & attended at least 7 f2f)	nr
Jacmon et al, 2009[8]	See BT	9/9 (100%)	See BT	3/9 (33.3%)	See BT	6/9 (67%)	6/9 (67%)
Kemmeren et al, 2019[9]	See BT	200/231 (87%)	nr	21/200 (10.5%)	See BT	179/200 (89.5%) criteria: 3 FTF; started 3 OL & spent >1h online	nr
Kenter et al, 2013[10]	104/104 (100%)	55/55 (100%) Criteria: at least 1 OL + F2F	13/104 (12.5%)	19/55 (34.5%)	unclear (WL data nr)	36/55 (65.5%) completed at least 3 sessions	10/55 pts (18.2%) criteria: completed all 5 sessions
Kenter et al, 2015[11]	na (selected records)	na	nr	nr	nr	nr	nr

**Authors**	**Overall Uptake - n(%)**	**BT - Uptake n(%)**	**Overall Dropouts- n(%)**	**BT Dropouts- n(%)**	**Overall adherence n(%)**	**BT adherence n(%)**	**BT Full treatment n (%)**
Kok et al, 2014[12]	193/212 (91%)	BT: 86/105 (82%)	84/193 (43.5%)	72/86 (84%)	unclear (WL data nr)	14/86 (16%) criteria: completed week 5	9/78 (11.5%) met intended usage (ie completed 8 exercises in 5 weeks)
Kooistra et al, 2016[13]	See BT	77% (7/9) (testing pts group)	See BT	0/7 (0%)	See BT	100% (7/7) completed a minimum of 30% of treatment	5/7 (71%)
Kooistra et al, 2019[14]	96/102 (94%) received at least 1 session	BT: 48/53 (90%)	20/96 (21%)	5/48 (10.5%)	65/96 (68%) Completion of 75% of 19 sessions (i.e. 14 sessions, regardless of ratio)	BT: 43/48 (89.5%)	7/54 (13%) (completed the full protocol within 10 weeks)
Kooistra et al, 2020[15]	76/92 (83%)	39/47 (83%)	9/76 (12%)	nr	nr	nr	nr
Lungu et al, 2020[16]	na (selected records)	na	nr	nr	nr	nr	nr
Ly et al, 2015[17]	88/93 (95%)	45/46 (98%)	3/88 (3.4%)	2/45 (4.5%)	81/88 (92%) (n of F2F competed)	BT: 43/45 (95.5%)	42/45 (93.3%)
Månsson et al, 2013[18]	15/15 (100 %) (selected group)	15/15 (100 %)	0/15 (0%)	0/15 (0%)	15/15 (100 %) (criteria unclear; assumed 100% as no treatment drop-off)	15/15 (100 %) (criteria unclear; assumed 100% as no treatment drop-off)	100%
Månsson et al, 2017[19]	42/54 (77%) (9 therapist s + 33 patients)	33/45 (73%) (patients only)	4/42 (9.5%) (9 therapists + 29 pts)	4/33 (12%) (patients only)	38/42 (90.5%)	29/33 (88%)	nr
Mol et al, 2018[20]	na (selected group)	45	See BT	7/45 (15.6%)	nr	nr	nr
Nakao et al, 2018[21]	40/40 100%	20/20 100%	nil	nil	40/40 (100%) (attending 8 sessions min)	20/20 (100%) (attending 8 sessions min)	20/20 (100%)
Romijn et al, 2021[22]	96/114 (84%) (selected sample)	44/52 (85%)	18/114 (16%)	10/44 (23%)	77/96 (80%) (defined as % of completed prescribed sessions)	34/44 (77%)	31/44 (70%)

Authors	Overall Uptake - n(%)	BT - Uptake n(%)	Overall Dropouts- n(%)	BT Dropouts- n(%)	Overall adherence n(%)	BT adherence n(%)	BT Full treatment n (%)
Tarp et al, 2022[23]	See BT	na<br><br>22 participants took surveys	See BT	6/22 (27%)	See BT	nr<br>16/22 (73%) answered outcome questionnaire	unclear
Thase et al, 2018[24]	154/154 (100%)	77/77 (100%)	29/154 (19%)	14/77 (18%)	125/154 (81%)	63/77 (82%) attended at least 2/3 sessions – BT: 8 F2F and 6 OL	nr
van de Wal et al, 2017[25]	85/88 (97%)	42/45 (93%)	11/85 (13%)	7/42 (17%)	74/85 (87%)	BT: 35/42 (83%) (used OL at least once)	28/42 (66%) completed all BT sessions
Vernmark et al, 2019[26]	141/151 (93%)	73/75 (97%)	nr (data on ctrl not known)	11/73 (15%)	nr	55/73 (75%) if cut-off = Completed at least 70%	nr
Witlox et al, 2021[27]	284/314 90%	137/150 (93%)	62/284 (21.9%)	37/137 (27%)	226/284 (80%) adherence= 3-4 F2F	100/137 (73%) adherence = 3-4 F2F	191/284 (67%) attended all F2F
Wu, Chen… & Lungu, 2021[28]	1496 (selected records)	1496 (selected records)	nr	262/1496 (17.5%)	See BT	1234/1496 (82.5%)	nr
Wu,Wickham, Chen, Chen & Lungu, 2021[29]	3401 (selected records)	3401 (selected records)	nr	nr	nr	nr	nr

KEY: BT: blended intervention group OL: online F2F: face-to-face pt: patients Tx: treatment
WL: waiting list nr: not reported nd: not defined

%**Uptake**: # of participants who completed at least 1 session divided by # randomised pts
%**Adherence**: # of participants who completed # of sessions considered minimal for reaching adherence to treatment completed divided by # of actual uptakers
%**Dropouts**: # participants who did not return posttreatment (T1) questionnaires divided by #of actual uptakers

---

References

1. Askjer S, Mathiasen K. The working alliance in blended versus face-to-face cognitive therapy for depression: A secondary analysis of a randomized controlled trial. Internet Interv; Sep 2021;25:100404. doi:10.1016/j.invent.2021.100404
2. Berger T, Krieger T, Sude K, Meyer B, Maercker A. Evaluating an e-mental health program ("deprexis") as adjunctive treatment tool in psychotherapy for depression: Results of a pragmatic randomized controlled trial. J Affect Disord; Feb 2018;227:455-462. doi:10.1016/j.jad.2017.11.021
3. Bisson JI, Ariti C, Cullen K, et al. Guided, internet based, cognitive behavioural therapy for post-traumatic stress disorder: pragmatic, multicentre, randomised controlled non-inferiority trial (RAPID). BMJ; Jun 16 2022;377:e069405. doi:10.1136/bmj-2021-069405
4. Cloitre M, Amspoker AB, Fletcher TL, et al. Comparing the Ratio of Therapist Support to Internet Sessions in a Blended Therapy Delivered to Trauma-Exposed Veterans: Quasi-experimental Comparison Study. JMIR Ment Health; Apr 27 2022;9(4):e33080. doi:10.2196/33080
5. Duffy D, Enrique A, Connell S, Connolly C, Richards D. Internet-Delivered Cognitive Behavior Therapy as a Prequel to Face-To-Face Therapy for Depression and Anxiety: A Naturalistic Observation. Front Psychiatry; 09 Jan 2020;10:902. 902. doi:10.3389/fpsyt.2019.00902

6. Etzelmueller A, Radkovsky A, Hannig W, Berking M, Ebert DD. Patient's experience with blended video- and internet based cognitive behavioural therapy service in routine care. Internet Interv; Jun 2018;12:165-175. doi:10.1016/j.invent.2018.01.003
7. Hoifodt RS, Lillevoll KR, Griffiths KM, et al. The clinical effectiveness of web-based cognitive behavioral therapy with face-to-face therapist support for depressed primary care patients: randomized controlled trial. Empirical Study; Interview; Quantitative Study. J Med Internet Res; Aug 5 2013;15(8):e153. doi:10.2196/jmir.2714
8. Jacmon J, Malouff JM, Taylor N. Treatment of major depression: Effectiveness of cognitive-behavioural therapy with an internet course as a central component. Empirical Study; Quantitative Study. E J Appl Psychol; 2009;5(2):1-8.
9. Kemmeren LL, van Schaik A, Smit JH, et al. Unraveling the Black Box: Exploring Usage Patterns of a Blended Treatment for Depression in a Multicenter Study. JMIR Ment Health; Jul 25 2019;6(7):e12707. doi:10.2196/12707
10. Kenter R, Warmerdam L, Brouwer-Dudokdewit C, Cuijpers P, van Straten A. Guided online treatment in routine mental health care: an observational study on uptake, drop-out and effects. BMC Psychiatry; Jan 31 2013;13:43. 43. doi:10.1186/1471-244X-13-43
11. Kenter RMF, van de Ven PM, Cuijpers P, et al. Costs and effects of Internet cognitive behavioral treatment blended with face-to-face treatment: Results from a naturalistic study. Internet Interv; 2015;2(1):77-83. doi:10.1016/j.invent.2015.01.001
12. Kok RN, van Straten A, Beekman AT, Cuijpers P. Short-term effectiveness of web-based guided self-help for phobic outpatients: randomized controlled trial. Empirical Study; Interview; Quantitative Study; Treatment Outcome. J Med Internet Res; Sep 29 2014;16(9):e226. doi:10.2196/jmir.3429
13. Kooistra LC, Ruwaard J, Wiersma JE, et al. Development and initial evaluation of blended cognitive behavioural treatment for major depression in routine specialized mental health care. Internet Interv; May 2016;4:61-71. doi:10.1016/j.invent.2016.01.003
14. Kooistra LC, Wiersma JE, Ruwaard J, et al. Cost and Effectiveness of Blended Versus Standard Cognitive Behavioral Therapy for Outpatients With Depression in Routine Specialized Mental Health Care: Pilot Randomized Controlled Trial. J Med Internet Res; Oct 29 2019;21(10):e14261. doi:10.2196/14261
15. Kooistra L, Ruwaard J, Wiersma J, van Oppen P, Riper H. Working Alliance in Blended Versus Face-to-Face Cognitive Behavioral Treatment for Patients with Depression in Specialized Mental Health Care. J Clin Med; Jan 27 2020;9(2)347. doi:10.3390/jcm9020347
16. Lungu A, Jun JJ, Azarmanesh O, Leykin Y, Chen CE. Blended Care-Cognitive Behavioral Therapy for Depression and Anxiety in Real-World Settings: Pragmatic Retrospective Study. J Med Internet Res; Jul 6 2020;22(7):e18723. e18723. doi:10.2196/18723
17. Ly KH, Topooco N, Cederlund H, et al. Smartphone-Supported versus Full Behavioural Activation for Depression: A Randomised Controlled Trial. PLoS One; 2015;10(5):e0126559. e0126559. doi:10.1371/journal.pone.0126559
18. Mansson KN, Skagius Ruiz E, Gervind E, Dahlin M, Andersson G. Development and initial evaluation of an Internet-based support system for face-to-face cognitive behavior therapy: a proof of concept study. J Med Internet Res; Dec 10 2013;15(12):e280. doi:10.2196/jmir.3031
19. Mansson KN, Klintmalm H, Nordqvist R, Andersson G. Conventional Cognitive Behavioral Therapy Facilitated by an Internet-Based Support System: Feasibility Study at a Psychiatric Outpatient Clinic. JMIR Res Protoc; Aug 24 2017;6(8):e158. doi:10.2196/resprot.6035
20. Mol M, Dozeman E, Provoost S, van Schaik A, Riper H, Smit JH. Behind the Scenes of Online Therapeutic Feedback in Blended Therapy for Depression: Mixed-Methods Observational Study. J Med Internet Res; May 3 2018;20(5):e174. doi:10.2196/jmir.9890
21. Nakao S, Nakagawa A, Oguchi Y, et al. Web-Based Cognitive Behavioral Therapy Blended With Face-to-Face Sessions for Major Depression: Randomized Controlled Trial. J Med Internet Res; Sep 21 2018;20(9):e10743. doi:10.2196/10743
22. Romijn G, Provoost S, Batelaan N, Koning J, van Balkom A, Riper H. Does it blend? Exploring therapist fidelity in blended CBT for anxiety disorders. Internet Interv; Sep 2021;25:100418. 100418. doi:10.1016/j.invent.2021.100418
23. Tarp K, Nielsen AS. Patient Perspectives on Videoconferencing-Based Treatment for Alcohol Use Disorders. Empirical Study; Interview; Qualitative Study. Alcohol Treat Q; Oct 2017;35(4):344-358. doi:10.1080/07347324.2017.1348785

24. Thase ME, Wright JH, Eells TD, et al. Improving the Efficiency of Psychotherapy for Depression: Computer-Assisted Versus Standard CBT. Am J Psychiatry; Mar 1 2018;175(3):242-250. doi:10.1176/appi.ajp.2017.17010089

25. van de Wal M, Thewes B, Gielissen M, Speckens A, Prins J. Efficacy of Blended Cognitive Behavior Therapy for High Fear of Recurrence in Breast, Prostate, and Colorectal Cancer Survivors: The SWORD Study, a Randomized Controlled Trial. J Clin Oncol; Jul 1 2017;35(19):2173-2183. doi:10.1200/JCO.2016.70.5301

26. Vernmark K, Hesser H, Topooco N, et al. Working alliance as a predictor of change in depression during blended cognitive behaviour therapy. Cogn Behav Ther; Jul 2019;48(4):285-299. doi:10.1080/16506073.2018.1533577

27. Witlox M, Garnefski N, Kraaij V, et al. Blended Acceptance and Commitment Therapy Versus Face-to-face Cognitive Behavioral Therapy for Older Adults With Anxiety Symptoms in Primary Care: Pragmatic Single-blind Cluster Randomized Trial. J Med Internet Res; Mar 26 2021;23(3):e24366. e24366. doi:10.2196/24366

28. Wu MS, Chen SY, Wickham RE, O'Neil-Hart S, Chen C, Lungu A. Outcomes of a Blended Care Coaching Program for Clients Presenting With Moderate Levels of Anxiety and Depression: Pragmatic Retrospective Study. JMIR Ment Health; Oct 21 2021;8(10):e32100. doi:10.2196/32100

29. Wu MS, Wickham RE, Chen SY, Chen C, Lungu A. Examining the Impact of Digital Components Across Different Phases of Treatment in a Blended Care Cognitive Behavioral Therapy Intervention for Depression and Anxiety: Pragmatic Retrospective Study. JMIR Form Res; Dec 17 2021;5(12):e33452. doi:10.2196/33452
