# Supplementary material for: Blended Psychological Therapy for the Treatment of Psychological Disorders in Adult Patients: Systematic Review and Meta-Analysis
Source: Interact J Med Res. 2024 Oct 29;13:e49660. doi: 10.2196/49660 (PMC11558224; doi:10.2196/49660)
Supplement: Multimedia Appendix 6 [file ijmr_v13i1e49660_app6.pdf]

Meta-analysis – BT vs Control Groups

1.1 Depression treatment outcomes – BT vs Control groups

- **Stats, ordered by effect size (the more negative, the larger the effect)**

Studyname	Statistics for each study						Weight (Random)		Residual (Random)			
	Standard error	Variance	Upper limit	Lower limit	Z-Value	p-Value	Relative weight	Relative weight	Std Residual	Std Residual	Std Residual	
Nakao et al, 2018	0.436	0.190	-1.835	-3.545	-6.164	0.000	9.17		-1.97			12.000
Askjer et al 2021	0.292	0.085	-1.613	-2.757	-7.487	0.000	10.74		-1.47			12.000
Høifødt et al, 2013	0.220	0.049	-1.082	-1.946	-6.870	0.000	11.42		-0.58			7.000
van de Wal et al 2017	0.229	0.052	-0.653	-1.550	-4.813	0.000	11.35		-0.00			12.000
Romijn G, et al., 2021	0.243	0.059	-0.471	-1.425	-3.898	0.000	11.22		0.21			15.000
Kooistra et al 2019	0.242	0.059	-0.459	-1.409	-3.856	0.000	11.22		0.23			10.000
Berger et al, 2018	0.250	0.062	-0.259	-1.238	-2.999	0.003	11.15		0.48			12.000
Thase et al. 2018	0.162	0.026	0.101	-0.532	-1.334	0.182	11.88		1.26			16.000
Bisson et al, 2022	0.167	0.028	0.327	-0.327	0.000	1.000	11.85		1.57			16.000
Pooled	0.074	0.006	-0.651	-0.943	-10.722	0.000						

1.2 Depression treatment outcomes – BT vs Control groups

- **Forest Plot, ordered by effect size (the more negative, the larger the effect)**

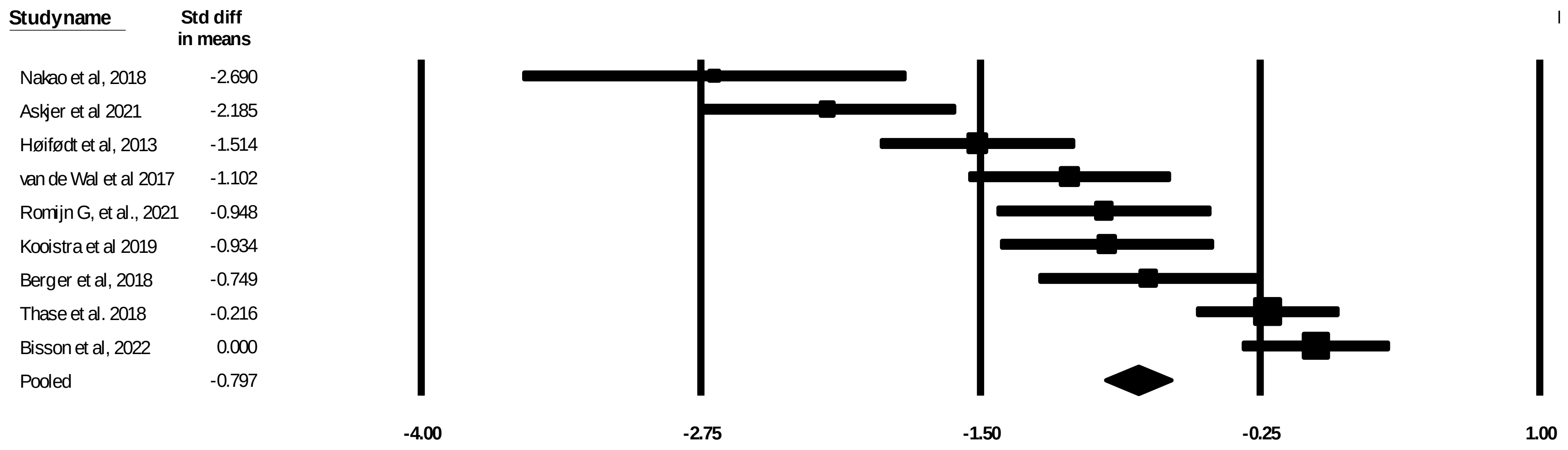

2 Depression treatment outcomes – BT vs Control groups – Funnel Plot

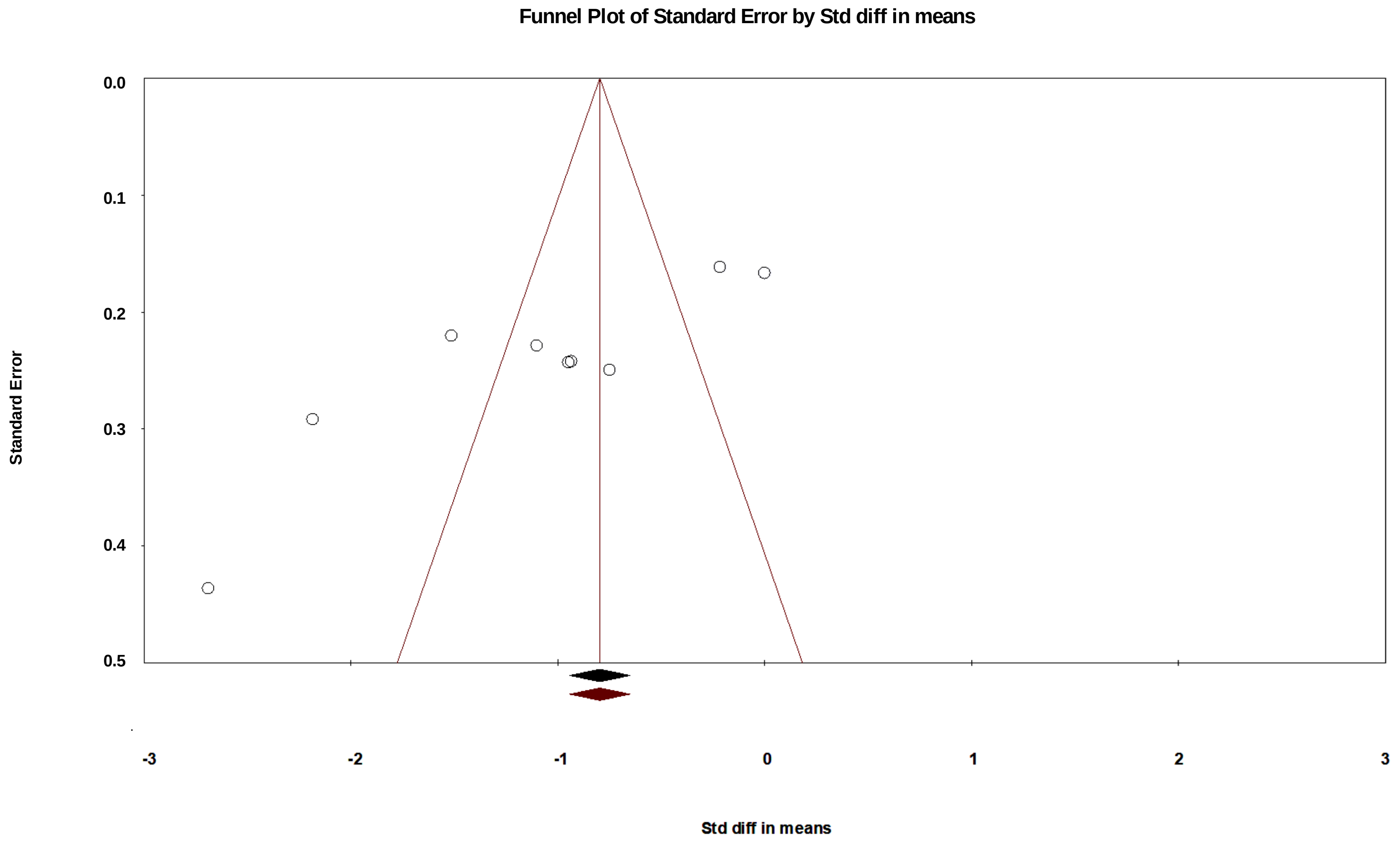

3.1 Anxiety treatment outcomes – BT vs Control groups

- Stats; ordered by author name (alphabetically)

Study name	Statistics for each study						
	Std diff in means	Standard error	Variance	Lower limit	Upper limit	Z-Value	p-Value
Berger et al, 2018	-0.116	0.242	0.058	-0.589	0.358	-0.479	0.632
Bisson et al, 2022	-0.041	0.168	0.028	-0.371	0.288	-0.246	0.806
Høifødt et al, 2013	-0.101	0.194	0.038	-0.482	0.280	-0.520	0.603
Romijn G, et al., 2021							
van de Wal et al 2017	-0.110	0.230	0.053	-0.560	0.341	-0.477	0.633
Pooled	-0.318	0.215	0.046	-0.738	0.103	-1.481	0.139
Prediction Interval	-0.126	0.091	0.008	-0.305	0.053	-1.380	0.167

3.2 Anxiety treatment outcomes – BT vs Control groups

- Forest Plot; ordered by author name (alphabetically)

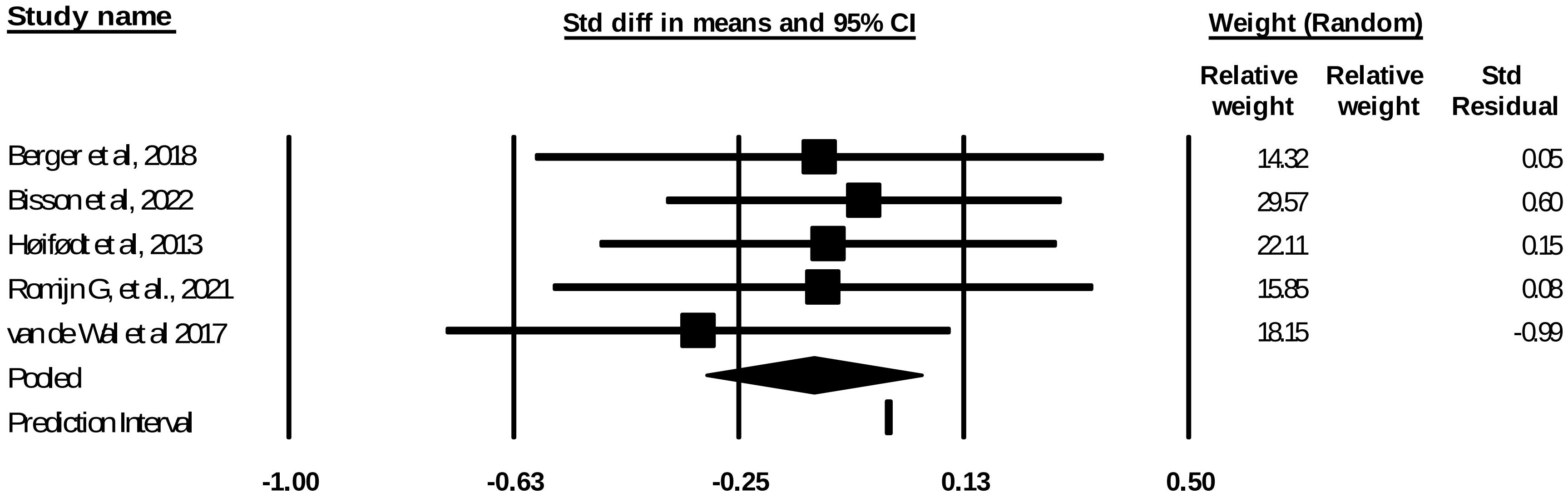

4. Anxiety treatment outcomes – BT vs Control groups – Funnel Plot

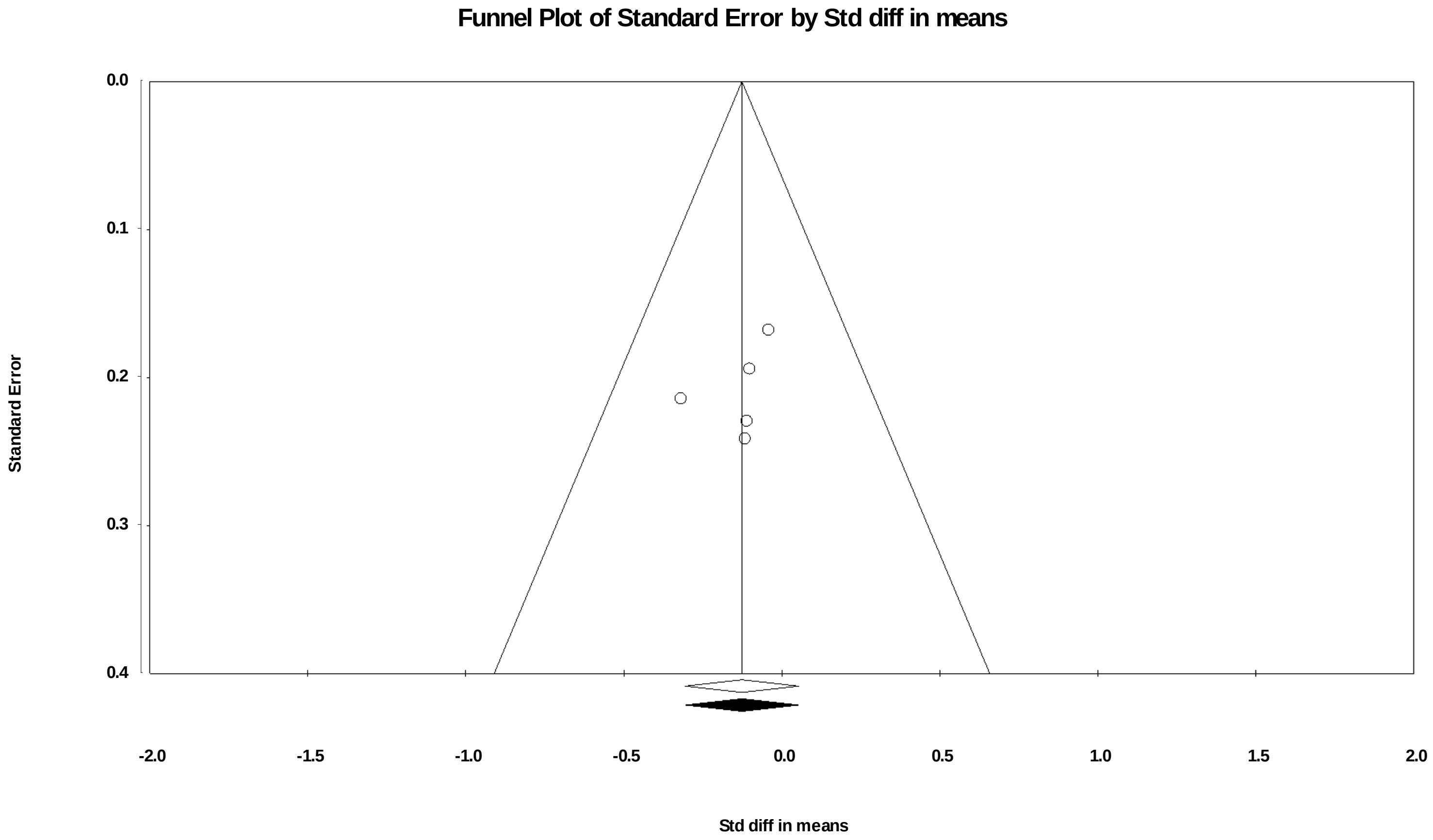

Subgroup Meta-analysis – BT structure vs effect size (ES) outcomes

5. Depression - Stats - ES per design; ordered by design (core vs supplementary), then by effect size (the more negative, the larger the effect)

Study	Mean - F2F	Mean - Online	Group by	Model	Statistics per study						
				Random	Std diff in means	Standard error	Variance	Upper limit	Lower limit	Z-Value	p-Value
Vernmark et al, 2019	4	10	Core		-0.81	1.97E-02	3.89E-04	-0.77	-0.84	-40.90	0.00
Høifødt et al, 2013	7.2	3.8	Core		-0.67	2.83E-02	8.03E-04	-0.62	-0.73	-23.81	0.00
Nakao et al, 2018	11.65	11.65	Core		-0.67	2.92E-02	8.55E-04	-0.61	-0.72	-22.75	0.00
Witlox et al, 2021	3.5	nd	Core		-0.65	4.27E-02	1.82E-03	-0.57	-0.73	-15.24	0.00
Romijn G, et al., 2021	6.7	6	Core		-0.61	3.12E-02	9.75E-04	-0.55	-0.67	-19.43	0.00
Thase et al. 2018	11	8.1	Core		-0.51	1.21E-02	1.47E-04	-0.48	-0.53	-41.94	0.00
Askjer et al 2021	5	4	Core		-0.45	0.02887	8.34E-04	-0.39	-0.51	-15.64	0.00
Kenter et al. 2013	nd	5	Core		-0.44	1.41E-02	1.99E-04	-0.41	-0.47	-31.13	0.00
Kok et al., 2014	nd	nd	Core		-0.41	2.32E-02	5.38E-04	-0.37	-0.46	-17.86	0.00
Mol et al, 2018	7.1	6.3	Core		-0.14	9.47E-03	8.97E-05	-0.12	-0.16	-14.43	0.00
			Core	Pooled	-0.53	7.71E-02	5.94E-03	-0.38	-0.69	-6.94	0.00
			Core	Pred Int	-0.53			0.05	-1.12		
Bisson et al, 2022	5	8	Supplementary		-1.18	3.37E-02	1.13E-03	-1.12	-1.25	-35.13	0.00
Lungu et al, 2020	5.2	nd	Supplementary		-1.07	2.20E-02	4.82E-04	-1.03	-1.11	-48.79	0.00
Kooistra et al 2016	7.11	8.6	Supplementary		-1.03	8.26E-02	6.82E-03	-0.87	-1.20	-12.53	0.00
Kooistra et al 2019	10	9.6	Supplementary		-1.01	2.57E-02	6.63E-04	-0.96	-1.06	-39.21	0.00
Ly et al, 2015	4	nd	Supplementary		-0.84	4.48E-02	2.00E-03	-0.76	-0.93	-18.85	0.00
Månsson et al, 2017	nd	14	Supplementary		-0.77	4.86E-02	2.36E-03	-0.68	-0.87	-15.89	0.00
Månsson et al, 2013	9	nd	Supplementary		-0.67	8.75E-02	7.66E-03	-0.50	-0.84	-7.62	0.00
Cloitre et al, 2022	7.5	7	Supplementary		-0.55	3.19E-02	1.02E-03	-0.49	-0.61	-17.30	0.00
Kooistra et al 2020	11.1	10.3	Supplementary		-0.49	5.12E-02	2.62E-03	-0.39	-0.59	-9.53	0.00
Berger et al, 2018	11.4	9	Supplementary		-0.36	2.63E-02	6.90E-04	-0.31	-0.41	-13.60	0.00
van de Wal et al 2017	6	nd	Supplementary		-0.31	6.96E-02	4.84E-03	-0.17	-0.45	-4.46	0.00
			Supplementary	Pooled	-0.75	0.098994	9.80E-03	-0.56	-0.95	-7.62	0.00
			Supplementary	Pred Int	-0.75			0.01	-1.52		
			Overall	Pooled	-0.62	6.08E-02	3.70E-03	-0.50	-0.74	-10.16	0.00
			Overall	Pred Int	-0.62			0.05	-1.28		

6. Depression - Forest Plot - ES per design, ordered by effect size (the more negative, the larger the effect)

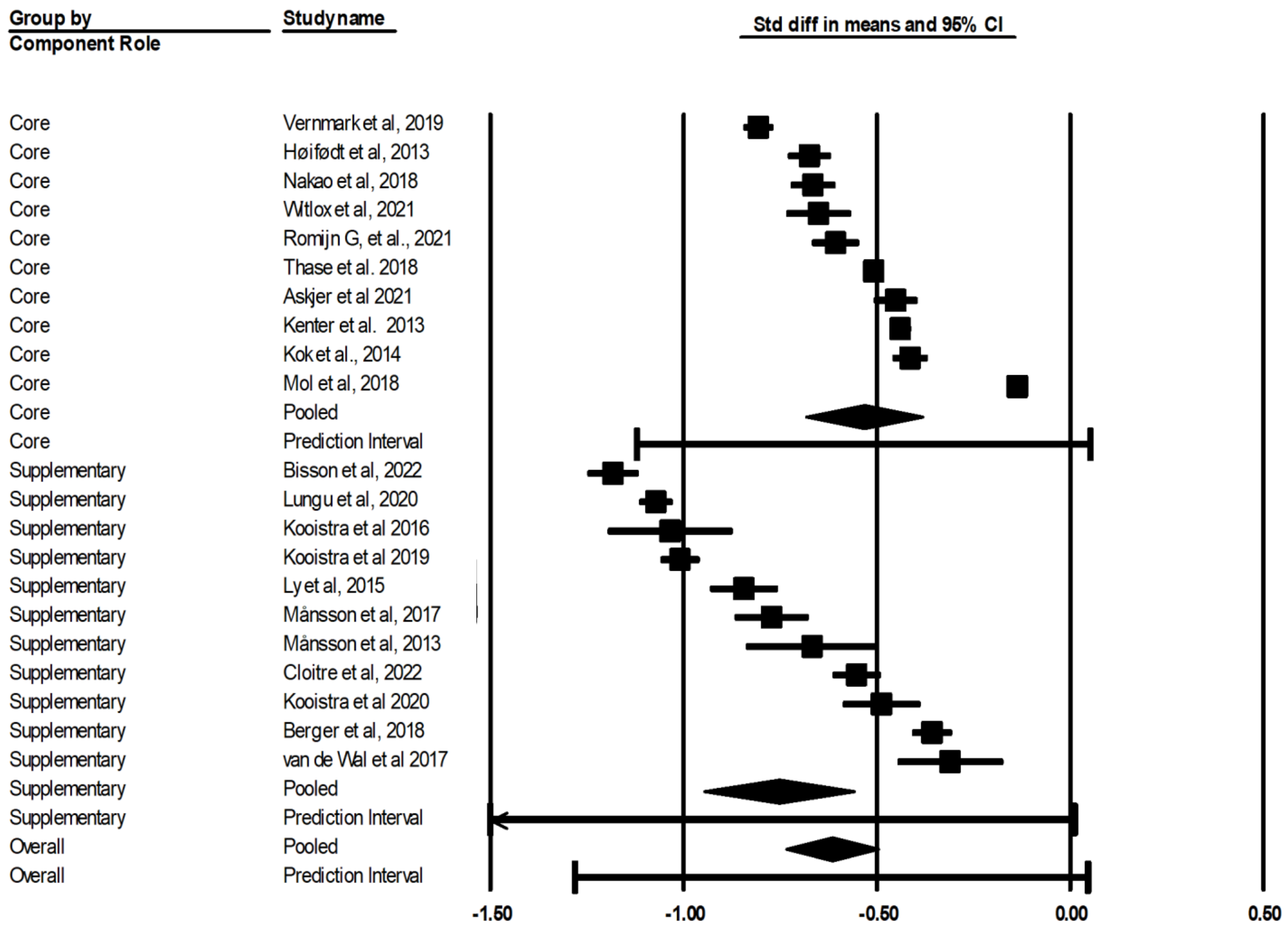

7. Depression - BT interventions - Funnel Plot

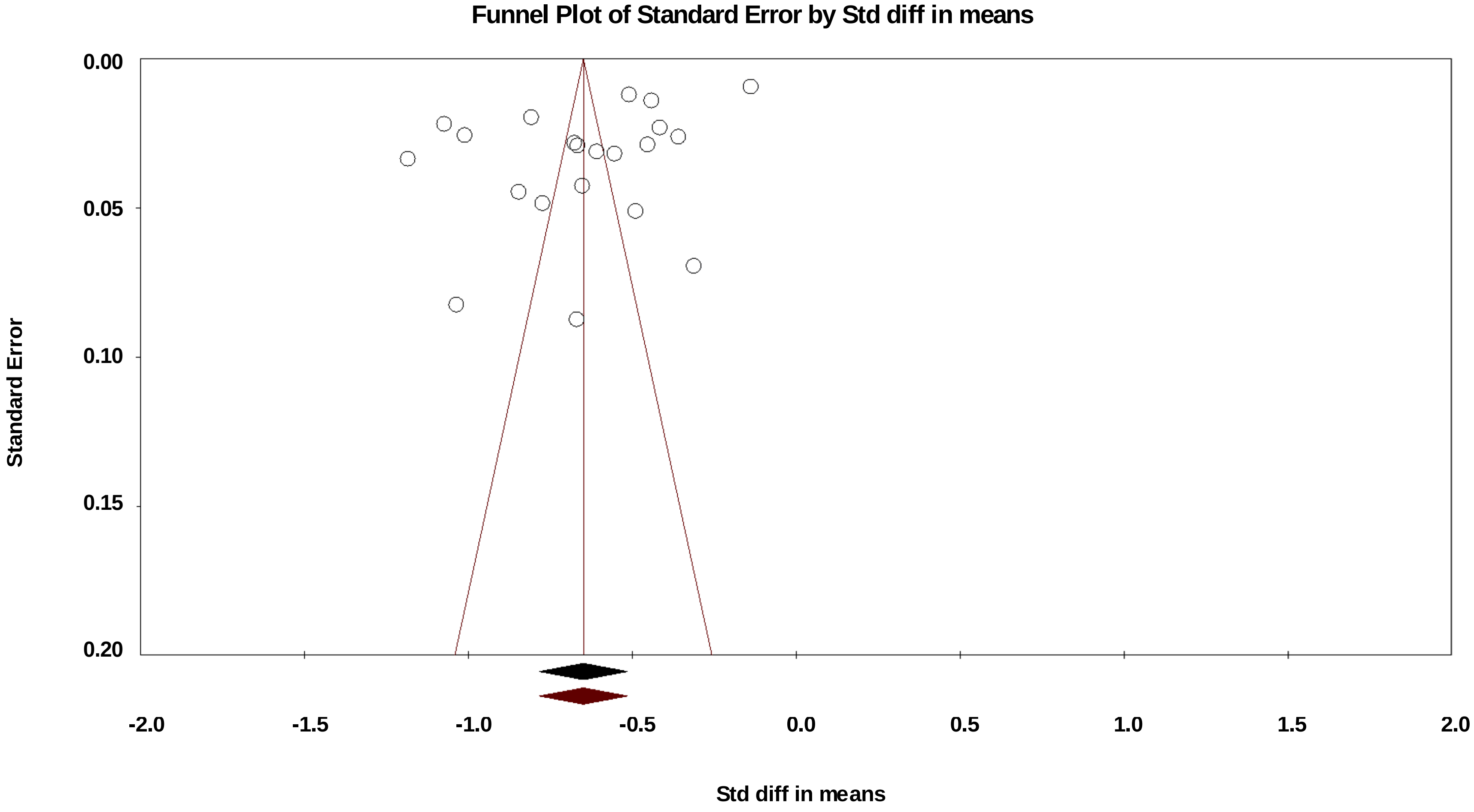

8. Anxiety - Stats per design

Component Role	Study name	Std diff in means	Standard error	Variance	Lower limit	Upper limit	Z-Value	p-Value
		Statistics for each study						
Core	Witlox et al, 2021	-0.926	0.078	0.006	-1.079	-0.772	-11.814	0.000
Core	Romijn G, et al., 2021	-0.888	0.075	0.006	-1.034	-0.741	-11.888	0.000
Core	Kenter et al. 2013	-0.749	0.045	0.002	-0.837	-0.661	-16.678	0.000
Core	Høifødt et al, 2013	-0.335	0.061	0.004	-0.456	-0.215	-5.458	0.000
Core	Kok et al., 2014	-0.112	0.015	0.000	-0.141	-0.084	-7.686	0.000
	Pooled	-0.599	0.193	0.037	-0.978	-0.220	-3.100	0.002
	Prediction Interval	-0.599			-2.094	0.896		
Supplementary	Bisson et al, 2022	-1.427	0.032	0.001	-1.490	-1.365	-44.596	0.000
Supplementary	Månsson et al, 2013	-1.275	0.208	0.043	-1.682	-0.867	-6.129	0.000
Supplementary	Lungu et al, 2020	-1.266	0.030	0.001	-1.325	-1.207	-41.803	0.000
Supplementary	Kooistra et al 2016	-0.860	0.412	0.170	-1.667	-0.053	-2.089	0.037
Supplementary	Ly et al, 2015	-0.587	0.060	0.004	-0.705	-0.469	-9.755	0.000
Supplementary	van de Wal et al 2016	-0.552	0.062	0.004	-0.673	-0.430	-8.901	0.000
Supplementary	Berger et al, 2018	-0.537	0.063	0.004	-0.661	-0.414	-8.541	0.000
Supplementary	Månsson et al, 2017	-0.485	0.098	0.010	-0.677	-0.293	-4.942	0.000
	Pooled	-0.869	0.159	0.025	-1.180	-0.557	-5.472	0.000
	Prediction Interval	-0.869			-1.981	0.244		
	Pooled	-0.760	0.123	0.015	-1.000	-0.520	-6.196	0.000
	Prediction Interval	-0.760			-2.147	0.627		

9. Anxiety - Forest Plot per design

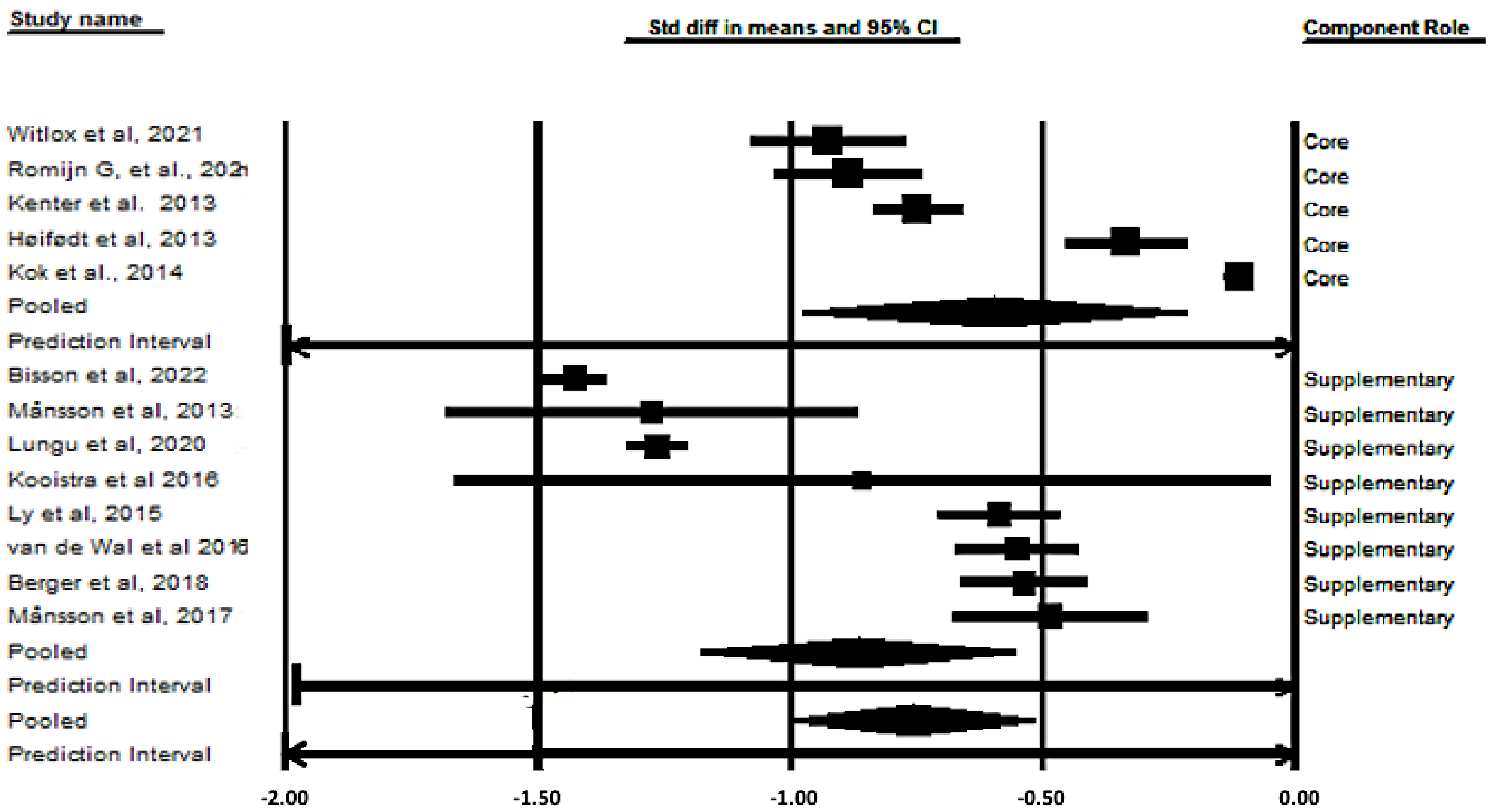

10. Anxiety - BT interventions - Funnel Plot

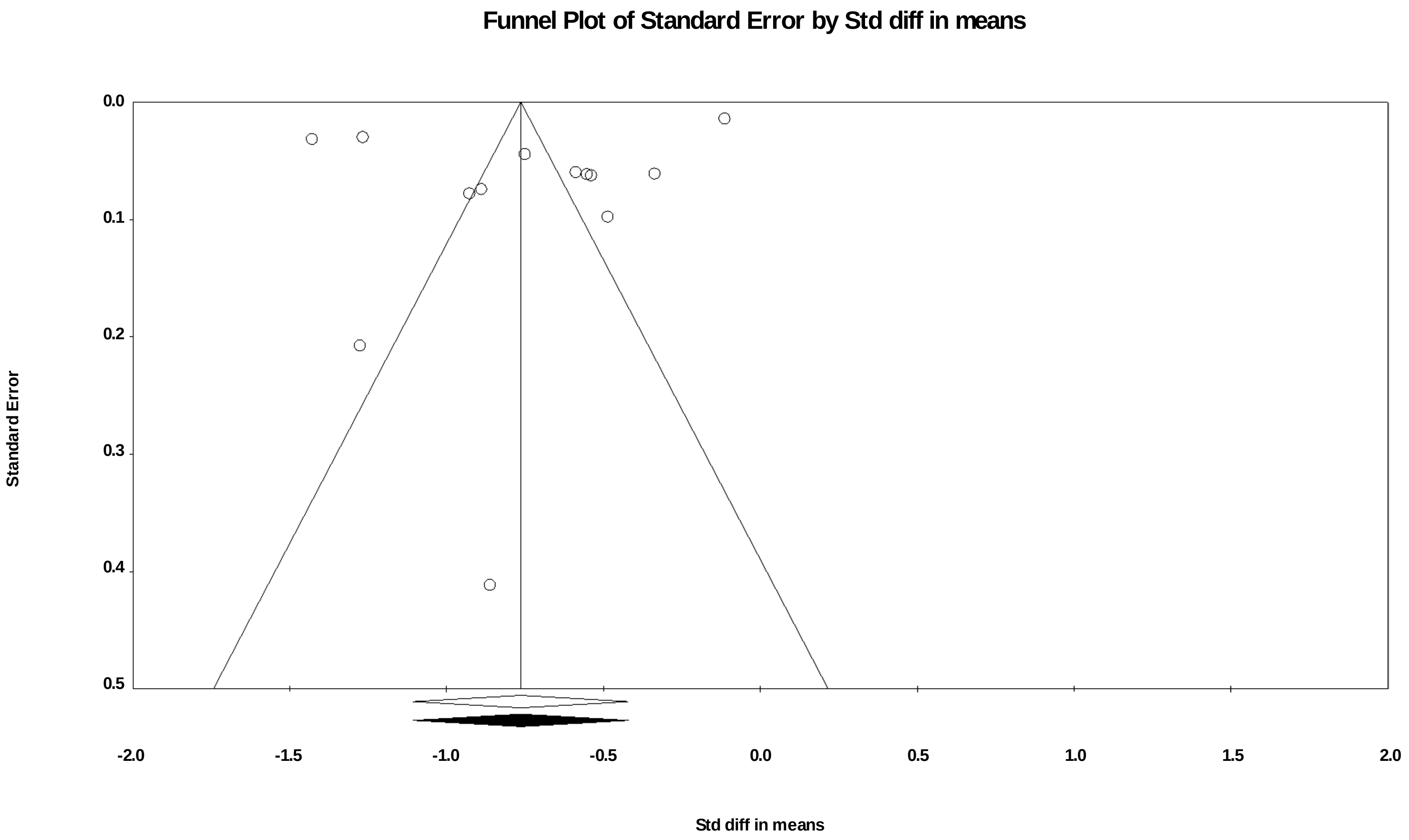

REFERENCES

Askjer, S., & Mathiasen, K. (2021). The working alliance in blended versus face-to-face cognitive therapy for depression: A secondary analysis of a randomized controlled trial. Internet Interv, 25, 100404. https://doi.org/10.1016/j.invent.2021.100404

Berger, T., Krieger, T., Sude, K., Meyer, B., & Maercker, A. (2018). Evaluating an e-mental health program ("deprexis") as adjunctive treatment tool in psychotherapy for depression: Results of a pragmatic randomized controlled trial. J Affect Disord, 227, 455-462. https://doi.org/10.1016/j.jad.2017.11.021

Bisson, J. I., Ariti, C., Cullen, K., Kitchiner, N., Lewis, C., Roberts, N. P., Simon, N., Smallman, K., Addison, K., Bell, V., Brookes-Howell, L., Cosgrove, S., Ehlers, A., Fitzsimmons, D., Foscarini-Craggs, P., Harris, S. R. S., Kelson, M., Lovell, K., McKenna, M., . . . Williams-Thomas, R. (2022). Guided, internet based, cognitive behavioural therapy for post-traumatic stress disorder: pragmatic, multicentre, randomised controlled non-inferiority trial (RAPID). BMJ, 377, e069405. https://doi.org/10.1136/bmj-2021-069405

Cloitre, M., Amspoker, A. B., Fletcher, T. L., Hogan, J. B., Jackson, C., Jacobs, A., Shammet, R., Speicher, S., Wassef, M., & Lindsay, J. (2022). Comparing the Ratio of Therapist Support to Internet Sessions in a Blended Therapy Delivered to Trauma-Exposed Veterans: Quasi-experimental Comparison Study. JMIR Ment Health, 9(4), e33080. https://doi.org/10.2196/33080

Hoifodt, R. S., Lillevoll, K. R., Griffiths, K. M., Wilsgaard, T., Eisemann, M., Waterloo, K., & Kolstrup, N. (2013). The clinical effectiveness of web-based cognitive behavioral therapy with face-to-face therapist support for depressed primary care patients: randomized controlled trial [Empirical Study; Interview; Quantitative Study]. J Med Internet Res, 15(8), e153. https://doi.org/10.2196/jmir.2714

Kenter, R., Warmerdam, L., Brouwer-Dudokdewit, C., Cuijpers, P., & van Straten, A. (2013). Guided online treatment in routine mental health care: an observational study on uptake, drop-out and effects. BMC Psychiatry, 13, 43, Article 43. https://doi.org/10.1186/1471-244X-13-43

Kok, R. N., van Straten, A., Beekman, A. T., & Cuijpers, P. (2014). Short-term effectiveness of web-based guided self-help for phobic outpatients: randomized controlled trial [Empirical Study; Interview; Quantitative Study; Treatment Outcome]. J Med Internet Res, 16(9), e226. https://doi.org/10.2196/jmir.3429

Kooistra, L., Ruwaard, J., Wiersma, J., van Oppen, P., & Riper, H. (2020). Working Alliance in Blended Versus Face-to-Face Cognitive Behavioral Treatment for Patients with Depression in Specialized Mental Health Care. J Clin Med, 9(2), Article 347. https://doi.org/10.3390/jcm9020347

Kooistra, L. C., Ruwaard, J., Wiersma, J. E., van Oppen, P., van der Vaart, R., van Gemert-Pijnen, J., & Riper, H. (2016). Development and initial evaluation of blended cognitive behavioural treatment for major depression in routine specialized mental health care. Internet Interv, 4, 61-71. https://doi.org/10.1016/j.invent.2016.01.003

Kooistra, L. C., Wiersma, J. E., Ruwaard, J., Neijenhuijs, K., Lokkerbol, J., van Oppen, P., Smit, F., & Riper, H. (2019). Cost and Effectiveness of Blended Versus Standard Cognitive Behavioral Therapy for Outpatients With Depression in Routine Specialized Mental Health Care: Pilot Randomized Controlled Trial. J Med Internet Res, 21(10), e14261. https://doi.org/10.2196/14261

Lungu, A., Jun, J. J., Azarmanesh, O., Leykin, Y., & Chen, C. E. (2020). Blended Care-Cognitive Behavioral Therapy for Depression and Anxiety in Real-World Settings: Pragmatic Retrospective Study. J Med Internet Res, 22(7), e18723, Article e18723. https://doi.org/10.2196/18723

Ly, K. H., Topooco, N., Cederlund, H., Wallin, A., Bergstrom, J., Molander, O., Carlbring, P., & Andersson, G. (2015). Smartphone-Supported versus Full Behavioural Activation for Depression: A Randomised Controlled Trial. PLoS ONE, 10(5), e0126559, Article e0126559. https://doi.org/10.1371/journal.pone.0126559

Mansson, K. N., Klintmalm, H., Nordqvist, R., & Andersson, G. (2017). Conventional Cognitive Behavioral Therapy Facilitated by an Internet-Based Support System: Feasibility Study at a Psychiatric Outpatient Clinic. JMIR Res Protoc, 6(8), e158. https://doi.org/10.2196/resprot.6035

Mansson, K. N., Skagius Ruiz, E., Gervind, E., Dahlin, M., & Andersson, G. (2013). Development and initial evaluation of an Internet-based support system for face-to-face cognitive behavior therapy: a proof of concept study. J Med Internet Res, 15(12), e280. https://doi.org/10.2196/jmir.3031

Mol, M., Dozeman, E., Provoost, S., van Schaik, A., Riper, H., & Smit, J. H. (2018). Behind the Scenes of Online Therapeutic Feedback in Blended Therapy for Depression: Mixed-Methods Observational Study. J Med Internet Res, 20(5), e174. https://doi.org/10.2196/jmir.9890

Nakao, S., Nakagawa, A., Oguchi, Y., Mitsuda, D., Kato, N., Nakagawa, Y., Tamura, N., Kudo, Y., Abe, T., Hiyama, M., Iwashita, S., Ono, Y., & Mimura, M. (2018). Web-Based Cognitive Behavioral Therapy Blended With Face-to-Face Sessions for Major Depression: Randomized Controlled Trial. J Med Internet Res, 20(9), e10743. https://doi.org/10.2196/10743

Romijn, G., Provoost, S., Batelaan, N., Koning, J., van Balkom, A., & Riper, H. (2021). Does it blend? Exploring therapist fidelity in blended CBT for anxiety disorders. Internet Interv, 25, 100418, Article 100418. https://doi.org/10.1016/j.invent.2021.100418

Thase, M. E., Wright, J. H., Eells, T. D., Barrett, M. S., Wisniewski, S. R., Balasubramani, G. K., McCrone, P., & Brown, G. K. (2018). Improving the Efficiency of Psychotherapy for Depression: Computer-Assisted Versus Standard CBT. Am J Psychiatry, 175(3), 242-250. https://doi.org/10.1176/appi.ajp.2017.17010089

van de Wal, M., Thewes, B., Gielissen, M., Speckens, A., & Prins, J. (2017). Efficacy of Blended Cognitive Behavior Therapy for High Fear of Recurrence in Breast, Prostate, and Colorectal Cancer Survivors: The SWORD Study, a Randomized Controlled Trial. J Clin Oncol, 35(19), 2173-2183. https://doi.org/10.1200/JCO.2016.70.5301

Vernmark, K., Hesser, H., Topooco, N., Berger, T., Riper, H., Luuk, L., Backlund, L., Carlbring, P., & Andersson, G. (2019). Working alliance as a predictor of change in depression during blended cognitive behaviour therapy. Cogn Behav Ther, 48(4), 285-299. https://doi.org/10.1080/16506073.2018.1533577

Witlox, M., Garnefski, N., Kraaij, V., de Waal, M. W. M., Smit, F., Bohlmeijer, E., & Spinhoven, P. (2021). Blended Acceptance and Commitment Therapy Versus Face-to-face Cognitive Behavioral Therapy for Older Adults With Anxiety Symptoms in Primary Care: Pragmatic Single-blind Cluster Randomized Trial. J Med Internet Res, 23(3), e24366, Article e24366. https://doi.org/10.2196/24366
